# Contribution of HIF-P4H isoenzyme inhibition to metabolism indicates major beneficial effects being conveyed by HIF-P4H-2 antagonism

**DOI:** 10.1016/j.jbc.2022.102222

**Published:** 2022-07-01

**Authors:** Joona Tapio, Riikka Halmetoja, Elitsa Y. Dimova, Joni M. Mäki, Anu Laitala, Gail Walkinshaw, Johanna Myllyharju, Raisa Serpi, Peppi Koivunen

**Affiliations:** 1 Biocenter Oulu, Faculty of Biochemistry and Molecular Medicine, Oulu Center for Cell-Matrix Research, University of Oulu, Oulu, Finland; 2 FibroGen Inc, San Francisco, California, USA; 3 Faculty of Medicine, University of Oulu, Oulu, Finland; 4 Biobank Borealis of Northern Finland, Oulu University Hospital, Oulu, Finland

**Keywords:** hypoxia, hypoxia-inducible factor (HIF), glucose metabolism, lipid metabolism, adipose tissue, AUC, area under the curve, CKD, chronic kidney disease, EPO, erythropoietin, GTT, glucose tolerance test, HDL, high-density lipoprotein, HIF, Hypoxia-inducible factor, HOMA-IR, homeostatic model assessment for insulin resistance, MEF, mouse embryonic fibroblast, OXPHOS, oxidative phosphorylation, P4H, prolyl 4-hydroxylase, WAT, white adipose tissue

## Abstract

Hypoxia-inducible factor (HIF) prolyl 4-hydroxylases (HIF-P4Hs 1–3) are druggable targets in renal anemia, where pan-HIF-P4H inhibitors induce an erythropoietic response. Preclinical data suggest that HIF-P4Hs could also be therapeutic targets for treating metabolic dysfunction, although the contributions of HIF-P4H isoenzymes in various tissues to the metabolic phenotype are inadequately understood. Here, we used mouse lines that were gene-deficient for HIF-P4Hs 1 to 3 and two preclinical pan-HIF-P4H inhibitors to study the contributions of these isoenzymes to the anthropometric and metabolic outcome and HIF response. We show both inhibitors induced a HIF response in wildtype white adipose tissue (WAT), liver, and skeletal muscle and alleviated metabolic dysfunction during a 6-week treatment period, but they did not alter healthy metabolism. Our data indicate that HIF-P4H-1 contributed especially to skeletal muscle and WAT metabolism and that its loss lowered body weight and serum cholesterol levels upon aging. In addition, we found HIF-P4H-3 had effects on the liver and WAT and its loss increased body weight, adiposity, liver weight and triglyceride levels, WAT inflammation, and cholesterol levels and resulted in hyperglycemia and insulin resistance, especially during aging. Finally, we demonstrate HIF-P4H-2 affected all tissues studied; its inhibition lowered body and liver weight and serum cholesterol levels and improved glucose tolerance. We found very few HIF target metabolic mRNAs were regulated by the inhibition of three isoenzymes, thus suggesting a potential for selective therapeutic tractability. Altogether, these data provide specifications for the future development of HIF-P4H inhibitors for the treatment of metabolic diseases.

The transcriptional hypoxia response is chiefly regulated by the hypoxia-inducible factor (HIF) system, in which three HIF prolyl 4-hydroxylase isoenzymes, HIF-P4Hs 1 to 3, (also known as PHDs 1–3 or EglN2, 1 and 3, respectively) provide the oxygen (O_2_)-sensing component for the system (1, 2). HIF-P4Hs are iron and 2-oxoglutarate–dependent dioxygenases that hydroxylate one or two proline residues in the HIFα subunit (3). The resulting 4-hydroxyproline residue acts as an earmark for pVHL binding and proteasomal degradation of HIFα (3). The hydroxylation is largely dependent on the cellular oxygenation status, as the HIF-P4Hs have a very low affinity (high *K*_*m*_ value) for O_2_, making them excellent sensors for hypoxia (3). The stabilized HIFα (one of the three isoforms, HIF1α-3α) forms a transcriptionally active dimer with HIFβ, which binds to the regulatory region of the HIF target gene and upregulates its transcription (1, 2). Altogether several hundred HIF target genes have been identified, with those for erythropoietin (EPO) and vascular endothelial growth factor being among the most studied, indicating that the HIF response aims to restore cellular oxygenation and O_2_ delivery by inducing erythropoiesis and angiogenesis (1, 2, 4). However, an even more central process to target in order to survive under hypoxia is energy metabolism, since mitochondrial oxidative phosphorylation (OXPHOS) is the most O_2_-consuming process in the cell (5). Thus the HIF target genes also upregulate genes that increase glucose intake and the non-oxygen-demanding glycolytic metabolism and downregulate OXPHOS, for instance (5, 6).

The HIF-P4Hs have been shown to be druggable targets, since several small-molecule inhibitors that typically compete with the binding of 2-oxoglutarate have been accepted since 2018 for the treatment of anemia in chronic kidney disease (CKD), first in Asia and very recently also in Europe (7). These antagonists that inhibit all three HIF-P4Hs upregulate endogenous renal EPO production (also hepatic EPO production in kidney-deficient patients) and support efficient iron metabolism, as many HIF target genes support iron intake and transfer (3, 8). *Via* EPO the HIF-P4H inhibitors also indirectly downregulate hepcidin (HAMP) levels, which are typically high in CKD and other inflammatory conditions, and inhibit cellular iron recycling (9). Interestingly, data from clinical trials with patients having CKD have shown that the HIF-P4H inhibitors also alter serum lipid values (10). Specifically, they reduce total cholesterol, non–high-density lipoprotein (HDL) cholesterol, and triglyceride levels (10). As these levels are elevated in dyslipidemia, a condition found in metabolic syndrome, these results could be desirable in a patient cohort suffering from metabolic dysfunction (11). The clinical data are supported by preclinical studies in which beneficial effects on lipid and glucose metabolism and inflammation have been reported with preclinical HIF-P4H inhibitors in several metabolic disease models (12, 13, 14, 15, 16), although the individual contributions of the HIF-P4H isoenzymes to the metabolic phenotype and the various tissues are not well understood. Beneficial effects of genetic inhibition of HIF-P4H-2 and HIF-P4H-1 on metabolism have been reported, but the data on HIF-P4H-3 have been contradictory (12, 16, 17, 18, 19, 20). We therefore used mouse lines gene modified for HIF-P4Hs 1 to 3 and two preclinical pan-HIF-P4H inhibitors, FG-4497 and FG-4539, to study the contributions of inhibition of the isoenzymes to the metabolic outcome in the key metabolic tissues. The resulting data will be of assistance in the future development of therapeutics to treat obesity, metabolic syndrome, and fatty liver disease by exploiting the HIF pathway as a novel treatment strategy.

## Results

### HIF-P4H-1 loss has beneficial effects on metabolism upon aging, while HIF-P4H-3 deficiency has aggravating effects

We have shown earlier that adiposity, WAT inflammation, fasting blood glucose levels, fasting serum insulin levels, and homeostatic model assessment for insulin resistance (HOMA-IR) scores all increase significantly in WT mice fed normal chow *ad libitum* and housed in a standard cage at 1 year of age as compared with young animals (4–5 months of age), whereas mice that are hypomorphic for HIF-P4H-2 (*Hif-p4h-2*^*gt/gt*^) are protected from these increases (16). We therefore used aging up to 1 year as a challenge for studying potential metabolic dysfunction in *Hif-p4h-1* and *Hif-p4h-3* knockout (KO) male mice (Fig. S1).

**Figure 1:**
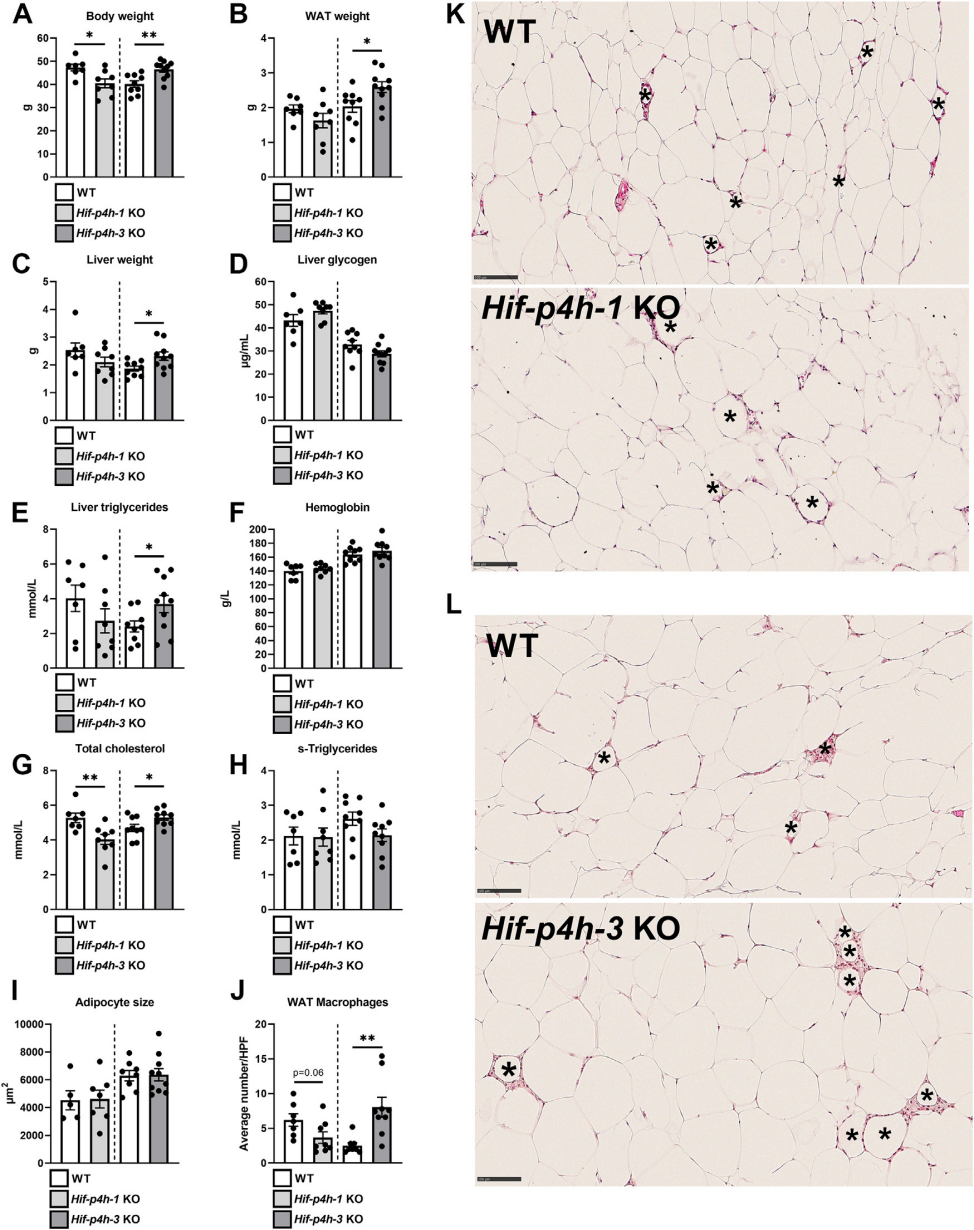
**HIF-P4H-1 deficiency has beneficial effects on metabolism upon aging, while HIF-P4H-3 deficiency has aggravating effects.***A*, body weight of 1-year-old *Hif-p4h-1* and *Hif-p4h-3* KO male mice compared with their C57BL/6N WT littermates (n = 7–9/group). *B,* weight of gonadal WAT. *C*, liver weight. *D* liver glycogen levels. *E,* liver triglyceride levels. *F,* hemoglobin levels. *G,* total cholesterol levels in serum. *H,* serum triglyceride levels. *I,* adipocyte size. *J,* average number of macrophage aggregates. *K,* macrophage aggregates in WT and *Hif-p4h-1* KO WAT. The latter image is reused in Fig. S2*A*. *L,* macrophage aggregates in WT and *Hif-p4h-3* KO WAT. Data are means ± SEM. ∗*p* ≤ 0.05, ∗∗*p* < 0.01. In K and L, macrophage aggregates in H&E (hematoxylin and eosin)-stained WAT sections are marked with an *asterisk* (∗). Figures *K* and *L* are 20× magnifications, the scale bar represents 100 μm. HPF, high power field; s, serum; WAT, white adipose tissue.

There were significant differences in body weight between the aged *Hif-p4h-1* KO and *Hif-p4h-3* KO mice at sacrifice and their WT littermates, the former having lower and the latter higher body weight (Fig. 1*A*). The *Hif-p4h-3* KO mice also had significantly more gonadal WAT and heavier livers than their WT littermates, whereas the tendency for lower values in the *Hif-p4h-1* KO mice relative to WT did not reach significance (Fig. 1, *B* and *C*). No difference in liver glycogen levels were detected between the *Hif-p4h-1* KO or *Hif-p4h-3* KO mice and their WT littermates (Fig. 1*D*), but the *Hif-p4h-3* KO mice had higher liver triglyceride levels than their WT counterparts (Fig. 1*E*). There were no significant differences in blood hemoglobin (Hb) levels between the aged *Hif-p4h-1* KO or *Hif-p4h-3* KO mice and their WT littermates (Fig. 1*F*), but the aged *Hif-p4h-1* KO mice had lower serum total cholesterol levels than their WT littermates and the *Hif-p4h-3* KO mice had higher levels (Fig. 1*G*). No differences in serum triglyceride levels were detected between the *Hif-p4h-1* or *3* KO mice and their littermates (Fig. 1*H*). The histologically analyzed adipocyte size did not differ between the aged *Hif-p4h-1* KO or *Hif-p4h-3* KO mice and WT, respectively, but there was an almost significant reduction in the number of macrophage aggregates in the *Hif-p4h-1* KO WAT and a significant increase in the *Hif-p4h-3* KO WAT (Figs. 1,*I*–*L* and S2).

**Figure 2:**
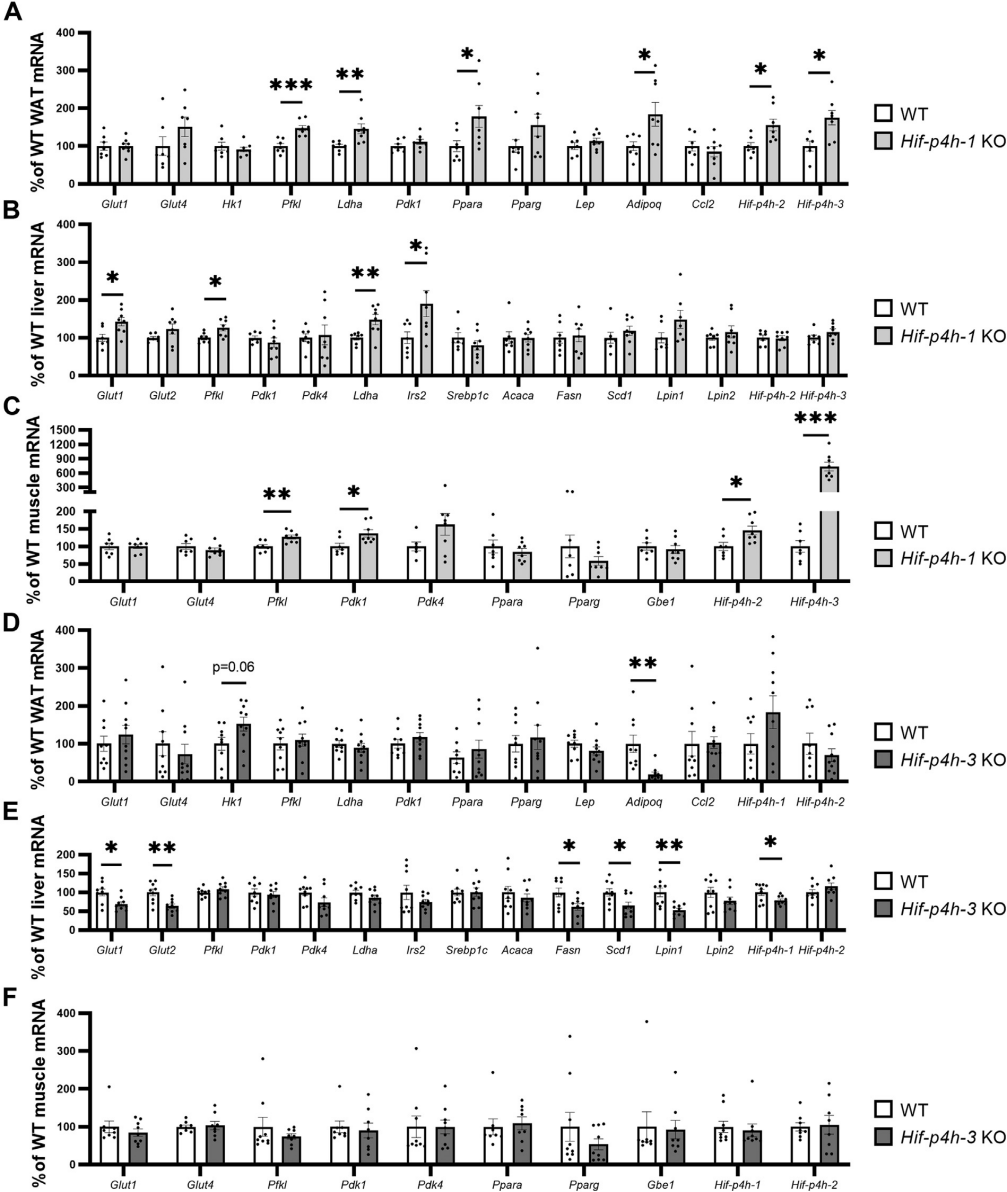
**Expression levels of the key metabolic HIF target mRNAs and certain other mRNAs in WAT, liver, and skeletal muscle of 1-year-old *Hif-p4h-1* and *Hif-p4h-3* KO mice by comparison with their C57BL/6N WT littermates.** Individual mRNA expressions are presented as percentages relative to the WT average (normalized to 100%) of the set mRNA level (n = 7–9/group). *A, Hif-p4h-1* KO WAT mRNA levels. *B, Hif-p4h-1* KO liver mRNA levels. *C, Hif-p4h-1* KO skeletal muscle mRNA levels. *D, Hif-p4h-3* KO WAT mRNA levels. *E, Hif-p4h-3* KO liver mRNA levels. *F Hif-p4h-3* KO skeletal muscle mRNA levels. All mRNAs were studied relative to β-actin protein mRNA. Data are means ± SEM. ∗*p* ≤ 0.05, ∗∗*p* < 0.01, ∗∗∗*p* < 0.001. Acaca, acetyl-CoA carboxylase α; Adipoq, adiponectin; Ccl2, chemokine ligand 2; Fasn, fatty acid synthase; Gbe1, 1,4-α-glucan branching enzyme 1; Glut1, glucose transporter 1; Glut2, glucose transporter 2; Glut4, glucose transporter 4; Hif-p4h-1-3, hypoxia-inducible factor prolyl-4 hydroxylase 1 to 3; Hk1, hexokinase 1; Irs2, insulin receptor substrate 2; Ldha, lactate dehydrogenase a; Lep, leptin; Lpin1, lipin-1; Lpin2, lipin-2; Ppara, peroxisome proliferator-activated receptor α; Pdk1, pyruvate dehydrogenase kinase; Pdk4, pyruvate dehydrogenase kinase 4; Pfkl, phosphofructokinase liver type; Pparg, peroxisome proliferator-activated receptor γ; Scd1, stearoyl-CoA desaturase-1; Srebp1c, sterol regulatory element-binding protein 1; WAT, white adipose tissue.

We next analyzed HIF1α/2α protein levels and the expression levels of the key metabolic HIF target and some other selected mRNAs in the WAT, liver, and skeletal muscle of the aged *Hif-p4h-1* KO and *Hif-p4h-3* KO mice by comparison with their WT littermates. Stabilization of HIF1α in WAT and skeletal muscle was detected by Western blotting in *Hif-p4h-1* KO mice (Fig. S3). The *Hif-p4h-1* KO tissues showed upregulation of glucose transporter 1 (*Glut1*, *Slc2a1*) mRNA in the liver, phosphofructokinase l (*Pfkl*)—the central regulator of glycolysis—in all the tissues studied, lactate dehydrogenase (*Ldha*) in WAT and liver, pyruvate dehydrogenase kinase 1 (*Pdk1*) in skeletal muscle, peroxisome proliferator-activated receptor α (*Ppara*) and adiponectin (*Adipoq*) in WAT, and insulin receptor substrate 2 (*Irs2*) in the liver (Fig. 2, *A*–*C*). Interestingly, there was also upregulation of the HIF target *Hif-p4h-2* and *Hif-p4h-3* mRNAs in WAT and skeletal muscle of the *Hif-p4h-1* KO mice as compared with WT (Fig. 2, *A* and *C*), which could suggest a compensatory attempt to overcome the *Hif-p4h-1* loss and indicate an important role for isoenzyme 1 in WAT and skeletal muscle.

No stabilization of HIF1α or HIF2α was detected (data not shown) and none of the glycolytic HIF target mRNAs were significantly upregulated in any of the *Hif-p4h-3* KO tissues studied (Fig. 2, *D*–*F*), but *Adipoq* mRNA was significantly downregulated in WAT and mRNAs for *Glut1* (*Slc2a1*), *Glut2* (*Slc2a2*), stearoyl-CoA desaturase-1 (*Scd1*), fatty acid synthase (*Fasn*), lipin-1 (*Lpin1*), and *Hif-ph4-1* were downregulated in the livers of the *Hif-p4h-3* KO mice relative to their WT littermates (Fig. 2, *D* and *E*). Downregulation of the central fatty acid synthesis mRNAs would suggest that the higher liver weight of the aged *Hif-p4h-3* KO mice did not stem from higher lipogenic activity.

In order to evaluate the contribution of the mRNA levels to the observed differences in the metabolic outcome, association analyses were carried out. These indicated a negative association between the WAT glycolytic HIF target mRNAs, *Ppara*, *Adipoq*, *Hif-p4h-2* and *Hif-p4h-3* mRNA, and body weight, WAT and liver weight, liver triglyceride and serum total cholesterol levels, and the WAT macrophage count in the *Hif-p4h-1* strain (Figs.S4*A*–S9*A*), suggesting that the increased levels observed in these mRNAs in the *Hif-p4h-1* KO WAT contributed to the favorable changes in several of these metabolic parameters (Fig. 1). Similarly, a negative association between WAT *Adipoq* mRNA levels and body weight, liver weight, and the WAT macrophage count was seen in the *Hif-p4h-3* strain, suggesting that the observed downregulation of its mRNA levels contributed to the detrimental outcome reflected in these parameters in the *Hif-p4h-3* KO mice (Figs. S4*B*, S6*B* and S9*B*). The negative associations between hepatic *Glut1*, *Glut2,* and *Hif-p4h-1* mRNA levels and body weight and WAT weight, between hepatic *Glut1* mRNA and total cholesterol levels, and between hepatic *Glut2* mRNA levels and the WAT macrophage count in the *Hif-p4h-3* strain (Figs. S4*B*, S5*B*, S7*B* and S9*B*) suggest a link between lower levels of these mRNAs in the *Hif-p4h-3* KO mice and their increased adiposity and WAT inflammation (Fig. 1).

### Small-molecule pan-HIF-P4H inhibitors mediate the effects on metabolic tissues and glucose and lipid metabolism

We then carried out a pilot experiment comparing the potential of two orally administered pan-HIF-P4H inhibitors, FG-4497, which has been used previously in preclinical studies (12, 16), and FG-4539, to induce an HIF response in metabolic tissues. WT C57BL/6N male mice were given a single dose of FG-4539 (30 or 60 mg/kg) or FG-4497 (60 mg/kg) and sacrificed 6 h afterward (Fig. S1). Both inhibitors stabilized HIF1α and HIF2α in WAT at all doses, although significant variation between individuals was detected (Fig. S10*A*). Both inhibitors stabilized HIF1α in the liver with a 60-mg/kg dose, and FG-4539 also stabilized HIF2α, although significant variation between individuals was detected (Fig. S10*B*). In skeletal muscle both inhibitors at 60-mg/kg dose stabilized HIF1α, again significant variation between individuals was seen (Fig. S10*C*). Both inhibitors increased s-EPO levels markedly relative to the vehicle, and dose dependence was seen with FG-4539 (Fig. S10*D*). Upregulation of the metabolic HIF target mRNAs *Glut1* (*Slc2a1*), *Pfkl*, *Pdk1*, *Pparγ,* and *Irs2*, and that of *Ppara*, was noted in the WAT, liver, or skeletal muscle, but due to the small number of animals these effects did not reach significance (Fig. S10, *E*–*G*). Also, *Hif-p4h-2* mRNA was upregulated in the inhibitor-treated liver and *Hif-p4h-3* mRNA in the WAT and liver (Fig. S10, *E* and *F*). These data indicated no major differences between the two inhibitors and supported the study of both for their metabolic outcome in a longer setting.

**Figure 3:**
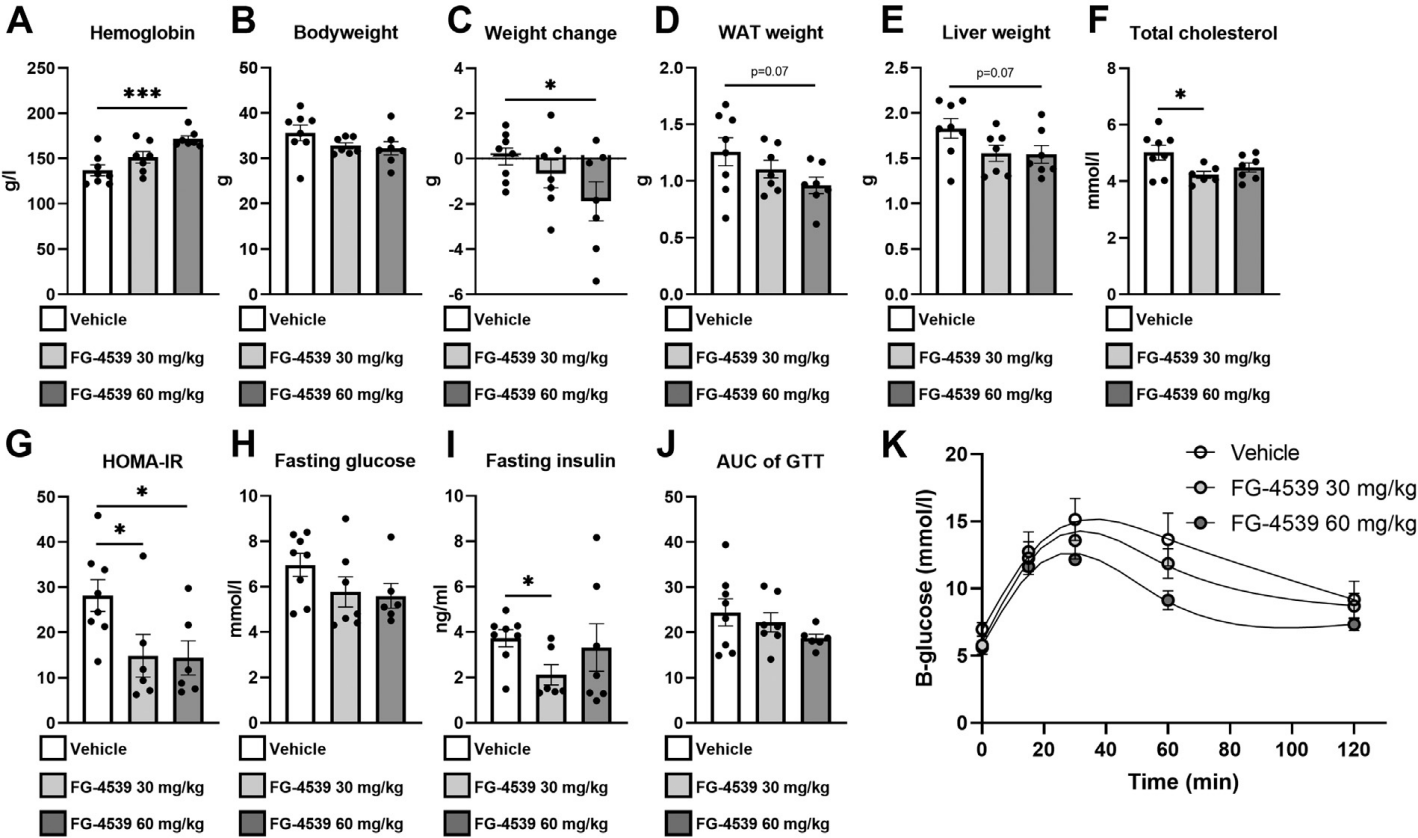
**A small-molecule pan-HIF-P4H inhibitormediateseffects on key metabolic tissues and glucose and lipid metabolism in 1-year-old C57BL/6N WT male mice.***A,* hemoglobin levels. *B,* body weight. *C,* weight change after 4 weeks of treatment. *D,* weight of gonadal WAT. *E,* liver weight. *F,* total cholesterol. *G,* HOMA-IR. *H,* fasting glucose. *I,* fasting insulin. *J,* AUC of GTT. *K,* GTT (n = 6–8/group). Data are means ± SEM. ∗*p* ≤ 0.05, ∗∗∗*p* <0.001. AUC, area under the curve; GTT, glucose tolerance test; HOMA-IR, homeostatic model assessment of insulin resistance; WAT, white adipose tissue.

We next administered FG-4539 3 times a week for 6 weeks to 6- to 7-month-old WT C57BL/6N males comparing 30 mg/kg and 60 mg/kg doses with the vehicle alone (Fig. S1). The mice were subjected to a glucose tolerance test (GTT) after 4 weeks of treatment and were sacrificed after 6 weeks of treatment. The mice receiving the inhibitor had a concentration-dependent increase in blood Hb levels at sacrifice, the higher dosage resulting in a ∼25% increase relative to the vehicle (Fig. 3*A*). There was an inhibitor dose-dependent decline in body weight, the weight change with the higher dosage being ∼−7% (∼−2 g) in 6 weeks (Fig. 3, *B* and *C*). There were also reductions of about 25% and 15% in the WAT and liver weights of the FG-4539-treated mice, respectively, although the 60-mg/kg dosage did not reach statistical significance (Fig. 3, *D* and *E*). The >15% decline in serum total cholesterol levels reached significance even with the lower FG-4539 dosage, however (Fig. 3*F*). There was a decline in fasting blood glucose levels in the FG-4539-treated mice, but unlike the reductions in fasting serum insulin levels or the HOMA-IR score, neither this nor the decline in the area under the curve (AUC) for GTT reached statistical significance (Fig. 3, *G*–*K*).

Interestingly, when FG-4539 was administered at a dose of 60 mg/kg to a WT male cohort with a different background (C57BL/6N/Sv129) and having before treatment a 35% lower body weight, 70% less WAT, and 35% lighter livers than the C57BL6/N cohort, no significant differences in the anthropometric or metabolic parameters were detected relative to the vehicle despite the increase in Hb levels (Figs. 3 and S11). These data suggest that the pan-HIF-P4H inhibitors only reverse adverse metabolic outcomes, which is important regarding the safety of their use, *e.g.*, for treating hyperglycemia.

### Analysis of adiposity and serum lipid levels of the HIF-P4H-1-3 isoenzyme-deficient mouse lines treated with pan-HIF-P4H inhibitors indicated some differences between the isoenzymes

We then administered 60 mg/kg FG-4539 or vehicle thrice a week for 6 weeks to 6- to 7-month-old *Hif-p4h-1* KO, *Hif-p4h-2*^*gt/gt*^, and *Hif-p4h-3* KO male mice and their WT male littermates (Fig. S1). A second cohort of the *Hif-p4h-3* KO male mice was treated similarly with FG-4497 for comparison (Fig. S1). In order to distinguish the role of each isoenzyme in the regulation of metabolism, four comparisons were made: (1) between vehicle-treated WT and vehicle-treated gene-deficient mice, to determine the contribution of genetic inhibition (total loss in the KO mice and tissue-specific downregulation in the *Hif-p4h-2*^*gt/gt*^ mice); (2) between inhibitor-treated WT and inhibitor-treated gene-deficient mice, to determine the role of genetic inhibition in addition to pharmacological inhibition of all isoenzymes; (3) between vehicle-treated WT and inhibitor-treated WT mice, to show the effect of pharmacological inhibition of all isoenzymes; and (4) between vehicle-treated gene-deficient mice and inhibitor-treated gene-deficient mice, which in the case of the KO mice would indicate the contribution of pharmacologic inhibition of the other isoenzymes but in the case of the *Hif-p4h-2*^*gt/gt*^ mice may also reflect the effect of further inhibition of HIF-P4H-2 by its pharmacological antagonist.

**Figure 4:**
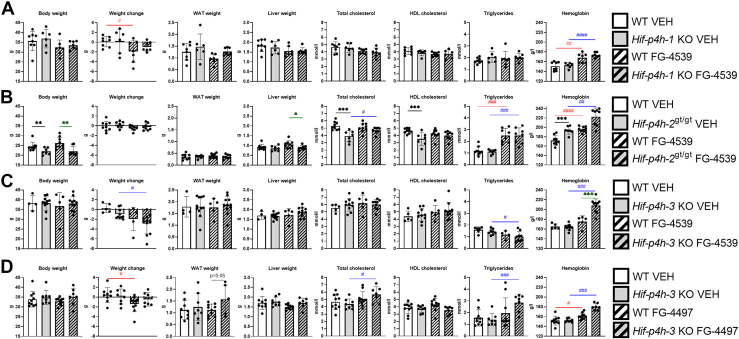
**Anthropometric measures, serum lipids, and hemoglobin levels of 7- to 8-month-old HIF-P4H-1-3 isoenzyme-deficient mouse lines treated with pan-HIF-P4H inhibitors.***A, Hif-p4h-1* KO mice and their WT littermates treated with FG-4539 or vehicle. *B, Hif-p4h-2*^gt/gt^ mice and their WT littermates treated with FG-4539 or vehicle. *C, Hif-p4h-3* KO mice and their WT littermates treated with FG-4539 or vehicle. *D, Hif-p4h-3* KO and WT littermates treated with FG-4497 or vehicle. (n=5–11/group). A *black asterisk* denotes a statistical difference between genotypes in vehicle-treated mice, a *green asterisk* a statistical difference between genotypes in FG-4539 or FG4497-treated mice, a *red hash* a statistical difference between vehicle and FG-4539 or FG4497-treated WT mice and a *blue hash* a statistical difference between vehicle and FG-4539 or FG4497-treated *Hif-p4h-1/3* KO or *Hif-p4h-2*^gt/gt^ mice. ∗ or # *p* ≤ 0.05, ∗∗ or ## *p* <0.01, ∗∗∗ or ### *p* < 0.001, #### *p* < 0.0001. HDL, high-density lipoprotein; VEH, vehicle-treated; WAT, white adipose tissue.

At sacrifice the *Hif-p4h-2*^*gt/gt*^ mice, which were in a different background from the others (C57BL6/N/Sv129) and were about 10 g lighter than the C57BL6/N mice, showed a genotype-mediated lower body weight that did not change with FG-4539 treatment (Fig. 4*B*). The inhibitor treatment–associated weight loss seen in the C57BL6/N WT mice relative to those receiving the vehicle was not observed in the FG-4539-treated *Hif-p4h-1* KO or FG-4497-treated *Hif-p4h-3* KO mice (Fig. 4, *A* and *D*). There was also a trend for an increased WAT weight in the FG-4497-treated *Hif-p4h-3* KO mice relative to WT (Fig. 4*D*), further supporting data from the aged *Hif-p4h-3* KO WAT (Fig. 1*B*). The only significant difference in liver weight was a decrease in the FG-4539-treated *Hif-p4h-2*^*gt/gt*^ mice relative to WT, which probably stemmed from pharmacological inhibition of HIF-P4H-2 in addition to the hypomorphic genetic inhibition (∼40% in the liver (16)), since the genetic inhibition of either HIF-P4H-1 or HIF-P4H-3 did not lower the liver weight (Fig. 4). The genotype-mediated lower serum total cholesterol and HDL cholesterol levels in the *Hif-p4h-2*^*gt/gt*^ mice were lost with FG-4539 treatment, and indeed, the former was slightly but significantly higher in the inhibitor-treated *Hif-p4h-2*^*gt/gt*^ mice than in those receiving the vehicle (Fig. 4*B*). These data suggest that simultaneous inhibition of all three isoenzymes does not result in the highest reduction in serum cholesterol levels. Serum triglyceride levels were increased with FG-4539 treatment independent of genotype in the C57BL6/N/Sv129 background but not in C57BL6/N (Fig. 4). As expected, Hb levels in the WT mice were increased by both pan-HIF-P4H inhibitors (Fig. 4). Inhibition of HIF-P4H-2 appeared to mediate the largest increase in Hb levels, and inhibition of HIF-P4H-3 also contributed to this (Fig. 4).

### Analysis of glucose tolerance and insulin sensitivity in the HIF-P4H-1-3 isoenzyme-deficient mouse lines treated with pan-HIF-P4H inhibitors indicated beneficial effects of HIF-P4H-2 inhibition but adverse effects of HIF-P4H-3 inhibition

**Figure 5:**
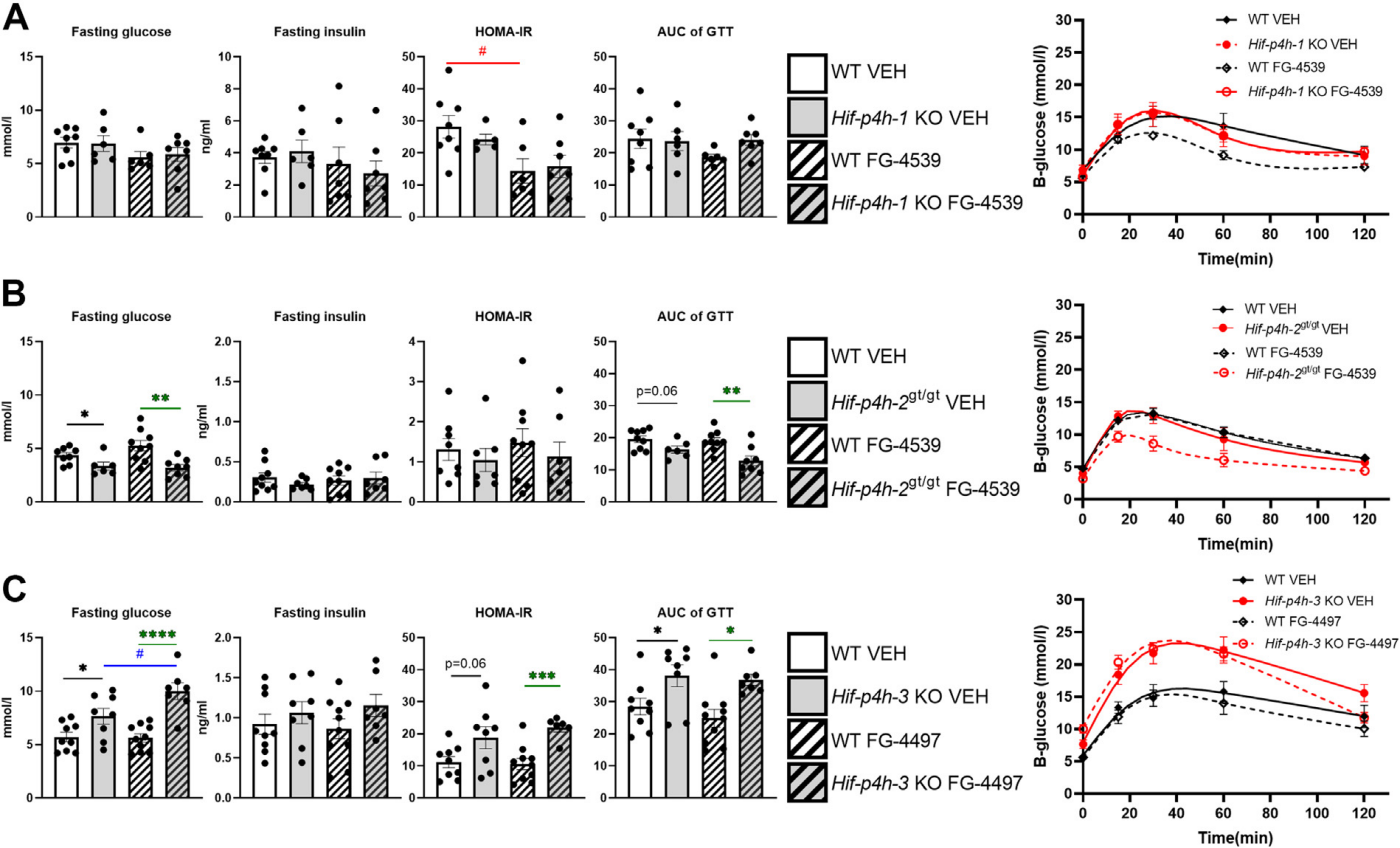
**Glucose tolerance and insulin sensitivity parameters of 7- to 8-month-old HIF-P4H-1-3 isoenzyme-deficient mouse lines treated with pan-HIF-P4H inhibitors.***A, Hif-p4h-1* KO mice and their WT littermates treated with FG-4539 or vehicle. *B, Hif-p4h-2*^gt/gt^ mice and their WT littermates treated with FG-4539 or vehicle. *C, Hif-p4h-3* KO mice and their WT littermates treated with FG-4497 or vehicle (n=5–11/group). A *black asterisk* denotes a statistical difference between genotypes in vehicle-treated mice, a *green asterisk* a statistical difference between genotypes in FG-4539 or FG4497-treated mice, a *red hash* a statistical difference between vehicle and FG-4539 or FG4497-treated WT mice, and a *blue hash* a statistical difference between vehicle and FG-4539 or FG4497-treated *Hif-p4h-1/3* KO or *Hif-p4h-2*^gt/gt^ mice. ∗ or #*p* ≤ 0.05, ∗∗*p* <0.01, ∗∗∗*p* < 0.001, ∗∗∗∗*p* < 0.0001. AUC, area under the curve; GTT, glucose tolerance test; HOMA-IR, homeostatic model assessment for insulin resistance; VEH, vehicle-treated.

When the mice were subjected to a GTT after 4 weeks of inhibitor treatment (Fig. S1) there were no differences in fasting glucose levels between the vehicle and FG-4539-treated *Hif-p4h-1* KO and WT littermates (Fig. 5*A*), while the *Hif-p4h-2*^*gt/gt*^ mice had genotype-mediated lower fasting glucose levels in both the vehicle and FG-4539-treated groups (Fig. 5*B*). The *Hif-p4h-3* KO mice had a genotype-mediated significant increase in fasting glucose levels, a trend for higher fasting insulin levels and significantly higher HOMA-IR levels in both the vehicle and FG-4497 treatments than did the WT mice (Fig. 5*B*). In agreement with the above data, the AUC of the GTT was genotype mediated and lower in the *Hif-p4h-2*^*gt/gt*^ mice and higher in the *Hif-p4h-3* KO mice than in the WT littermates, while no difference was seen in the *Hif-p4h-1* KO mice (Fig. 5). Altogether these data suggest that inhibition of HIF-P4H-1 does not contribute significantly to glucose intake and insulin sensitivity, whereas HIF-P4H-2 inhibition ameliorates these factors and HIF-P4H-3 inhibition exacerbates them.

### The metabolic HIF target and other genes in HIF-P4H-1-3 isoenzyme-deficient adipose tissue, liver, and skeletal muscle are regulated by pan-HIF-P4H inhibitors

Finally, we carried out large-scale analyses of the expression of mRNAs of metabolic HIF target genes and certain others in WAT, liver, and skeletal muscle of inhibitor- or vehicle-treated *Hif-p4h-1*/*3* KO and *Hif-p4h-2*^*gt/gt*^ mice by comparison with WT (Fig. S1). It should be noted that the experimental setting varied, in that the final dose of FG-4539 was administered to the *Hif-p4h-1* KO and *Hif-p4h-3* KO mice 24 h before sacrifice, whereas the *Hif-p4h-2*^*gt/gt*^ mice received their final dose of FG-4539 and the *Hif-p4h-3* KO mice that of FG-4497 6 h before sacrifice (Fig. S1). Depending on the half-life of the mRNAs this may have influenced the data.

**Figure 6:**
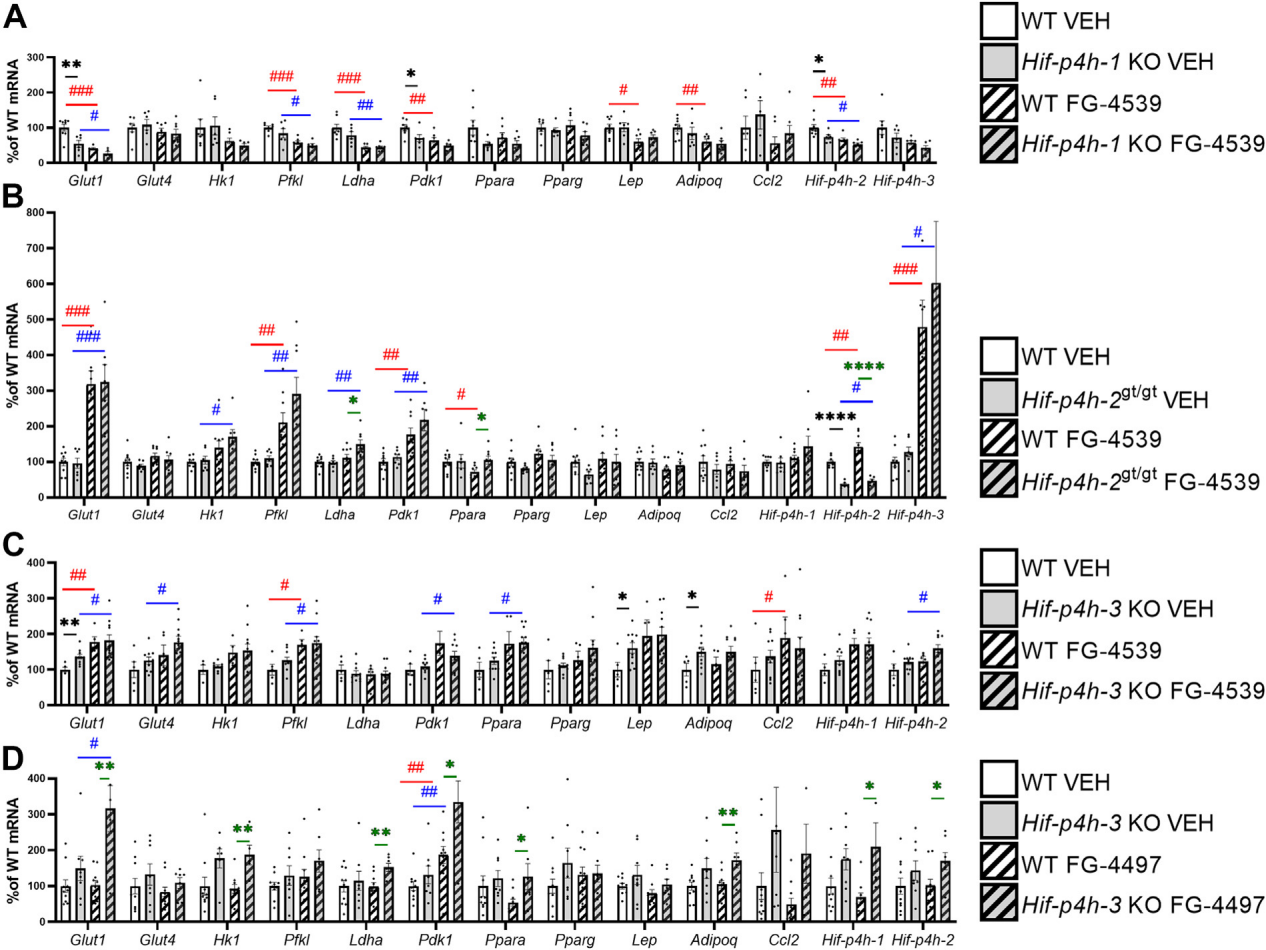
**Expression levels of the key metabolic HIF target mRNAs and certain other mRNAs in WAT of 7- to 8-month-old HIF-P4H-1-3 isoenzyme-deficient mouse lines treated with pan-HIF-P4H inhibitors.** Individual mRNA expressions are presented as percentages relative to the WT VEH average (normalized to 100%) of the set mRNA level. *A*, *Hif-p4h-1* KO mice and their WT littermates treated with FG-4539 or vehicle. *B*, *Hif-p4h-2*^gt/gt^ mice and their WT littermates treated with FG-4539 or vehicle. *C*, *Hif-p4h-3* KO mice and their WT littermates treated with FG-4539 or vehicle. *D*, *Hif-p4h-3* KO mice and their WT littermates treated with FG-4497 or vehicle (n=5–11/group). ∗ or #*p* ≤ 0.05, ∗∗ or ## *p* <0.01, ### *p* < 0.001, ∗∗∗∗*p* < 0.0001. A *black asterisk* denotes a statistical difference between genotypes in vehicle-treated mice, a *green asterisk* a statistical difference between genotypes in FG-4539 or FG4497-treated mice, a *red hash* a statistical difference between vehicle and FG-4539 or FG4497-treated WT mice, and a *blue hash* a statistical difference between vehicle and FG-4539 or FG4497-treated *Hif-p4h-1/3* KO or *Hif-p4h-2*^gt/gt^ mice. Adipoq, adiponectin; Ccl2, chemokine ligand 2; Glut1/4, glucose transporter 1/4; Hif-p4h-1-3, hypoxia-inducible factor prolyl-4 hydroxylase 1 to 3; Hk1, hexokinase 1; Ldha, lactate dehydrogenase a; Lep, leptin; Ppara, peroxisome proliferator-activated receptor α; Pdk1, pyruvate dehydrogenase kinase; Pfkl, phosphofructokinase l; Pparg, peroxisome proliferator-activated receptor γ; VEH, vehicle-treated; WAT, white adipose tissue.

The vehicle-treated mice showed genotype-mediated changes in very few mRNA levels in WAT, some of which differed from those affected in the WAT of the aged *Hif-p4h-1* KO and *Hif-p4h-3* KO (Figs. 6 and 2, *A* and *D*). Inhibitor treatment in addition to the genetic deficiency upregulated *Ldha* and *Ppara* mRNA in the *Hif-p4h-2*^*gt/gt*^ WAT compared with WT, and *Glut1* (*Slc2a1*), hexokinase 1 (*Hk1*), *Ldha*, *Pdk1*, *Ppara*, *Adipoq*, *Hif-p4h-1,* and *Hif-p4h-2* mRNAs in the FG-4497-treated *Hif-p4h-3* KO WAT (Fig. 6). Where FG-4539 treatment upregulated many glucose metabolism HIF target genes in the WAT of the WT littermates of the *Hif-p4h-2*^*gt/gt*^ and *Hif-p4h-3* KO mice, independent of the time when they received the final dose of the inhibitor, this treatment led to the opposite results in the WT littermates of the *Hif-p4h-1* KO mice, in which *Glut1* (*Slc2a1*), *Pfkl*, *Ldha*, *Pdk1,* and *Hif-p4h-2* mRNA levels were significantly downregulated compared with the vehicle 24 h after the final dosage (Fig. 6*A*). This difference may suggest a rebound effect in these animals following the inhibitor treatment. Interpretation of the contribution of inhibition of each HIF-P4H isoenzyme to the mRNA levels was further complicated by the fact that the knockdown level of HIF-P4H-2 in the WAT of the *Hif-p4h-2*^*gt/gt*^ mice was ∼60%, agreeing with earlier data (16). Altogether, the data regarding WAT do suggest that all the HIF-P4H isoenzymes contributed to the expression of the metabolic HIF target genes. Surprisingly, and in contrast to the data on the aged *Hif-p4h-3* KO WAT, several mRNAs such as *Glut1* (*Slc2a1*), *Hk1*, *Pdk1*, *Lep,* and *Adipoq* were largely regulated by inhibition of HIF-P4H-3 alone, while HIF-P4H-2 inhibition also contributed to the upregulation of *Ldha* and *Ppara* mRNA and inhibition of all the isoenzymes to *Pfkl* mRNA upregulation (Fig. 6). As for the inflammatory chemokine ligand 2 (Ccl2) mRNA, this was lowered by inhibition of HIF-P4H-2 but increased by inhibition of HIF-P4H-3 or HIF-P4H-1, although none of the effects reached statistical significance (Fig. 6).

**Figure 7:**
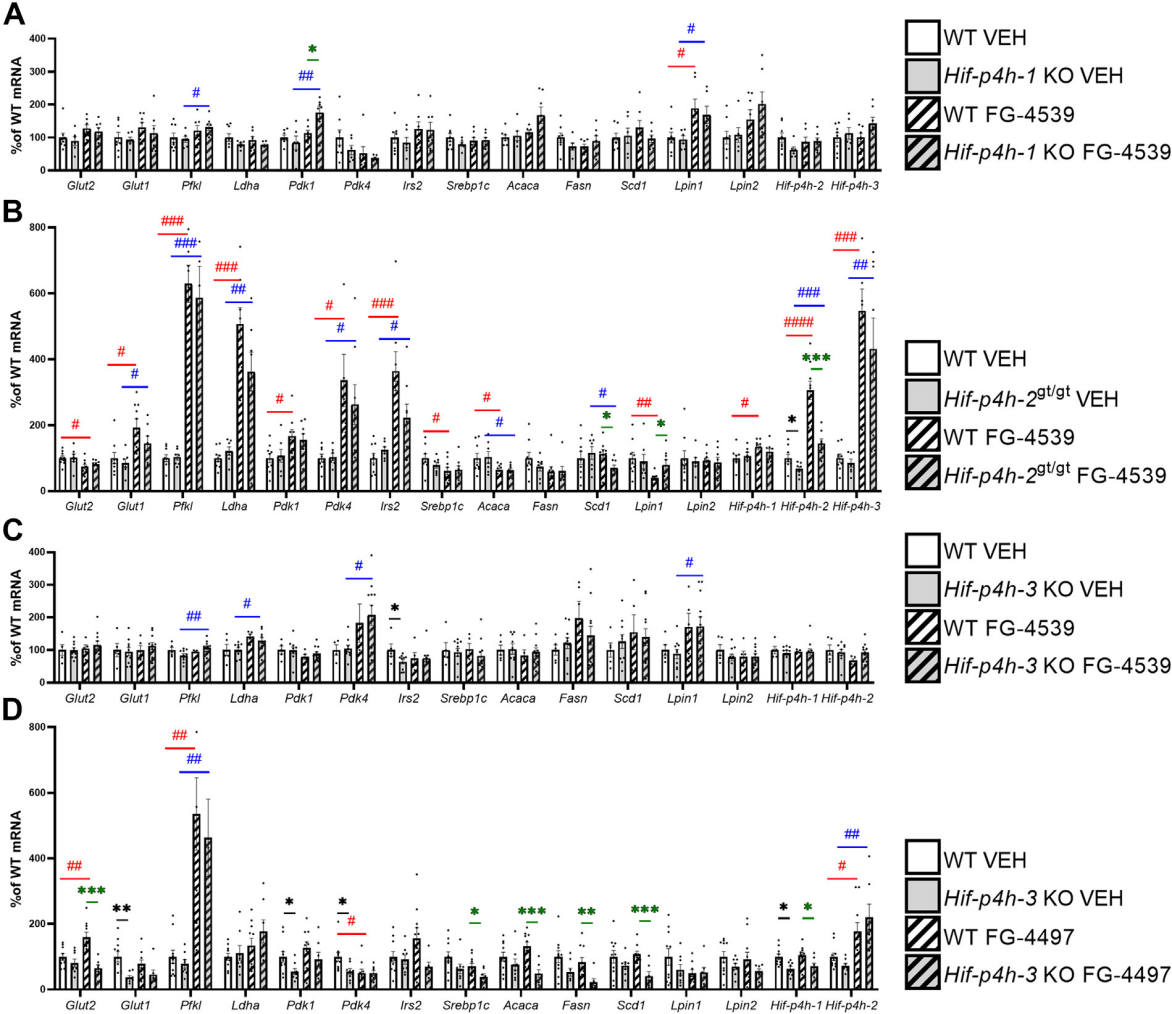
**Hepatic expression levels of the key metabolic HIF target mRNAs and certain other mRNAs in 7- to 8-month-old HIF-P4H-1-3 isoenzyme-deficient mouse lines treated with pan-HIF-P4H inhibitors.** Individual mRNA expressions are presented as percentages relative to the WT VEH average (normalized to 100%) of the set mRNA level. *A*, *Hif-p4h-1* KO mice and their WT littermates treated with FG-4539 or vehicle. *B*, *Hif-p4h-2*^gt/gt^ mice and their WT littermates treated with FG-4539 or vehicle. *C*, *Hif-p4h-3* KO mice and their WT littermates treated with FG-4539 or vehicle. *D*, *Hif-p4h-3* KO mice and their WT littermates treated with FG-4497 or vehicle. (n=5–11/group). ∗ or # *p* ≤ 0.05, ∗∗ or ## *p* < 0.01, ∗∗∗ or ### *p* < 0.001, #### *p* < 0.0001. A *black asterisk* denotes a statistical difference between genotypes in vehicle-treated mice, a *green asterisk* a statistical difference between genotypes in FG-4539 or FG4497-treated mice, a *red hash* a statistical difference between vehicle and FG-4539 or FG4497-treated WT mice, and a *blue hash* a statistical difference between vehicle and FG-4539 or FG4497-treated *Hif-p4h-1/3* KO or *Hif-p4h-2*^gt/gt^ mice. Acaca, acetyl-CoA carboxylase α; Fasn, fatty acid synthase; Glut1/2, glucose transporter 1/2; Hif-p4h-1-3, hypoxia-inducible factor prolyl-4 hydroxylase 1 to 3; Irs2, insulin receptor substrate 2; Ldha, lactate dehydrogenase a; Lpin1, lipin-1; Lpin2, lipin-2; Pdk1, pyruvate dehydrogenase kinase; Pdk4, pyruvate dehydrogenase kinase 4; Pfkl, phosphofructokinase l; Scd1, stearoyl-CoA desaturase-1; Screbp1c, sterol regulatory element-binding protein 1; VEH, vehicle-treated.

In the liver, the vehicle-treated *Hif-p4h-3* KO mice showed similar but not absolutely identical downregulation of glucose and lipid metabolism mRNAs to that seen in the aged mice (Figs. 7 and 2*E*). No genotype-mediated significant differences were detected in the mRNA levels studied in the vehicle-treated *Hif-p4h-2*^*gt/gt*^ liver (where the knockdown of HIF-P4H-2 was ∼40% of the WT level), agreeing with earlier data (16), or in the *Hif-p4h-1* KO liver relative to WT (Fig. 7). FG-4539 treatment upregulated *Pdk1* mRNA in *Hif-p4h-1* KO liver relative to WT and downregulated *Scd1* mRNA in the *Hif-p4h-2*^*gt/gt*^ liver (Fig. 7, *A* and *B*), while FG-4497 treatment downregulated *Glut2* (*Slc2a2*), sterol regulatory element-binding protein 1 (*Srebp1*), acetyl-CoA carboxylase α (*Acaca*), *Fasn*, *Scd1,* and *Hif-p4h-1* mRNA in the *Hif-p4h-3* KO liver (Fig. 7*D*). Both FG-4539 and FG-4497 upregulated glycolytic HIF target mRNAs and downregulated lipogenic mRNAs in the WT liver compared with the vehicle (Fig. 7). Although it is difficult to draw conclusions regarding the contribution of the inhibition of each of the HIF-P4H isoenzymes to mRNA levels in the liver, the data do suggest that inhibition of HIF-P4H-3 contributed to the downregulation of the glucose-regulated *Glut2* (*Slc2a2*) mRNA and also *Glut1* (*Slc2a1*) mRNA, while the inhibition of HIF-P4H-2 upregulated *Glut1* (*Slc2a1*) mRNA (Fig. 7). Upregulation of *Pfkl*, *Ldha*, *Pdk1*, *Pdk4,* and insulin sensitivity-increasing *Irs2* mRNAs was mediated by HIF-P4H-2 inhibition (Fig. 7), whereas downregulation of lipogenic *Srebp1c*, *Acaca,* and *Scd1* mRNAs was mediated by inhibition of HIF-P4H-2 and HIF-P4H-3 and the upregulation of *Hif-p4h-3* mRNA was largely mediated by HIF-P4H-2 inhibition (Fig. 7).

**Figure 8:**
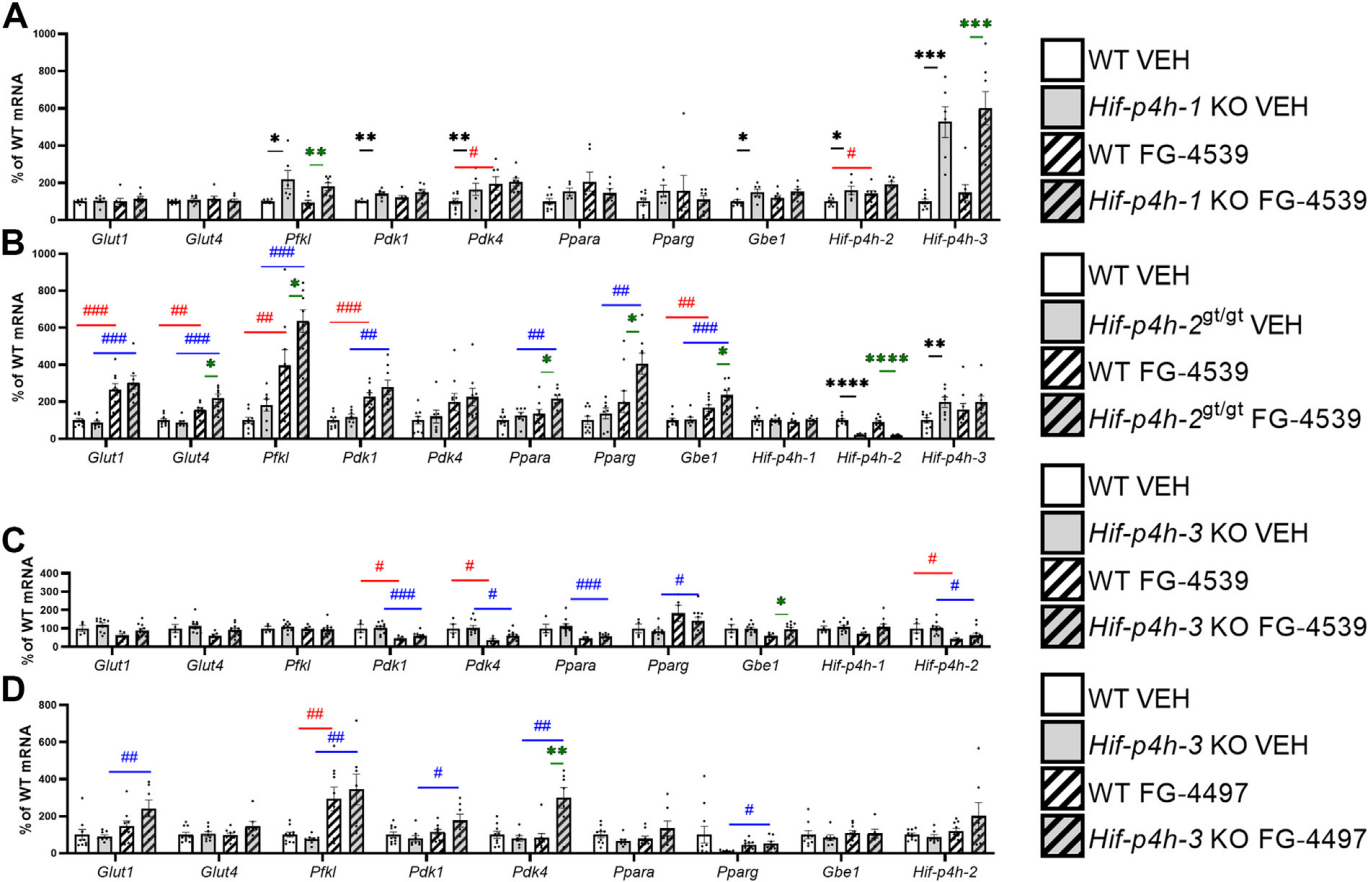
**Expression levels of the key metabolic HIF target mRNAs and certain other mRNAs in skeletal muscle of 7- to 8-month-old HIF-P4H-1-3 isoenzyme-deficient mouse lines treated with pan-HIF-P4H inhibitors.** Individual mRNA expressions are presented as percentages relative to the WT VEH average (normalized to 100%) of the set mRNA level. *A*, *Hif-p4h-1* KO mice and their WT littermates treated with FG-4539 or vehicle. *B*, *Hif-p4h-2*^gt/gt^ mice and their WT littermates treated with FG-4539 or vehicle. *C*, *Hif-p4h-3* KO mice and their WT littermates treated with FG-4539 or vehicle. *D*, *Hif-p4h-3* KO mice and their WT littermates treated with FG-4497 or vehicle (n=5–11/group). ∗ or # *p* ≤ 0.05, ∗∗ or ## *p* <0.01, ∗∗∗ or ### *p* < 0.001, ∗∗∗∗*p* < 0.0001. A *black asterisk* denotes a statistical difference between genotypes in vehicle-treated mice, a *green asterisk* a statistical difference between genotypes in FG-4539 or FG4497-treated mice, a *red hash* a statistical difference between vehicle and FG-4539 or FG4497-treated WT mice, and a *blue hash* a statistical difference between vehicle and FG-4539 or FG4497-treated *Hif-p4h-1/3* KO or *Hif-p4h-2*^gt/gt^ mice. Gbe1, 1,4-α-glucan branching enzyme 1; Glut1/4, glucose transporter ¼; Hif-p4h-1-3, hypoxia-inducible factor prolyl-4 hydroxylase 1 to 3; Ppara, peroxisome proliferator-activated receptor α; Pdk1, pyruvate dehydrogenase kinase; Pdk4, pyruvate dehydrogenase kinase 4; Pfkl, phosphofructokinase l; Pparg, peroxisome proliferator-activated receptor γ; VEH, vehicle-treated.

In skeletal muscle the vehicle-treated *Hif-p4h-1* KO mice compared with WT had an upregulation of the glycogenesis-regulating HIF target 1,4-α-glucan branching enzyme 1 (Gbe1) and *Hif-p4h-2/3* mRNAs similar to that seen in the aged *Hif-p4h-1* KO mice (Figs. 8*A* and 2*C*). It should be noted, however, that HIF-P4H-1 loss did not upregulate *Glut1/4* (*Slc2a1*/*4*) mRNA levels (Fig. 8). In the *Hif-p4h-2*^*gt/gt*^ skeletal muscle, where the knockdown of HIF-P4H-2 was about 80% of WT, as reported earlier (21), a genotype-mediated upregulation of *Hif-p4h-3* mRNA was detected in the vehicle-treated mice and the mRNAs for *Glut4* (*Slc2a4*), the key regulator of glucose intake; Pfkl; Ppara; and Pparg were significantly upregulated following FG-4539 treatment (Fig. 8*B*). In the *Hif-p4h-3* KO skeletal muscle, FG-4497 treatment upregulated *Gbe1* mRNA and FG-4539 treatment *Pdk4* mRNA relative to the vehicle (Fig. 8, *C* and *D*). Both inhibitors upregulated glucose intake and glycolytic metabolism mRNAs in WT skeletal muscle as compared with the vehicle, the effect being more widespread with FG-4539 than with FG-4497 (Fig. 8). Altogether, the skeletal muscle data suggested specificity in the regulation of the HIF target mRNAs by inhibition of the HIF-P4H isoenzymes, in that *Glut1/4* (*Slc2a1*/*4*) mRNAs were upregulated by inhibition of HIF-P4H-2, *Pfkl* by HIF-P4H-1/2, *Pdk1* by HIF-P4H-1, *Pdk4* by HIF-P4H-1/3, *Ppara* and *Pparg* by HIF-P4H-2, and *Gbe1* mRNA by that of all the isoenzymes (Fig. 8). The “compensatory” upregulation of the mRNAs for *Hif-p4h-2/3* in the *Hif-p4h-1* KO skeletal muscle and that for *Hif-p4h-3* in *Hif-p4h-2*^*gt/gt*^ may suggest that these isoenzymes play a key role in skeletal muscle (Fig. 8).

### HIF1α stabilization associates with decreased oxygen consumption and ATP production and increased glycolysis

**Figure 9:**
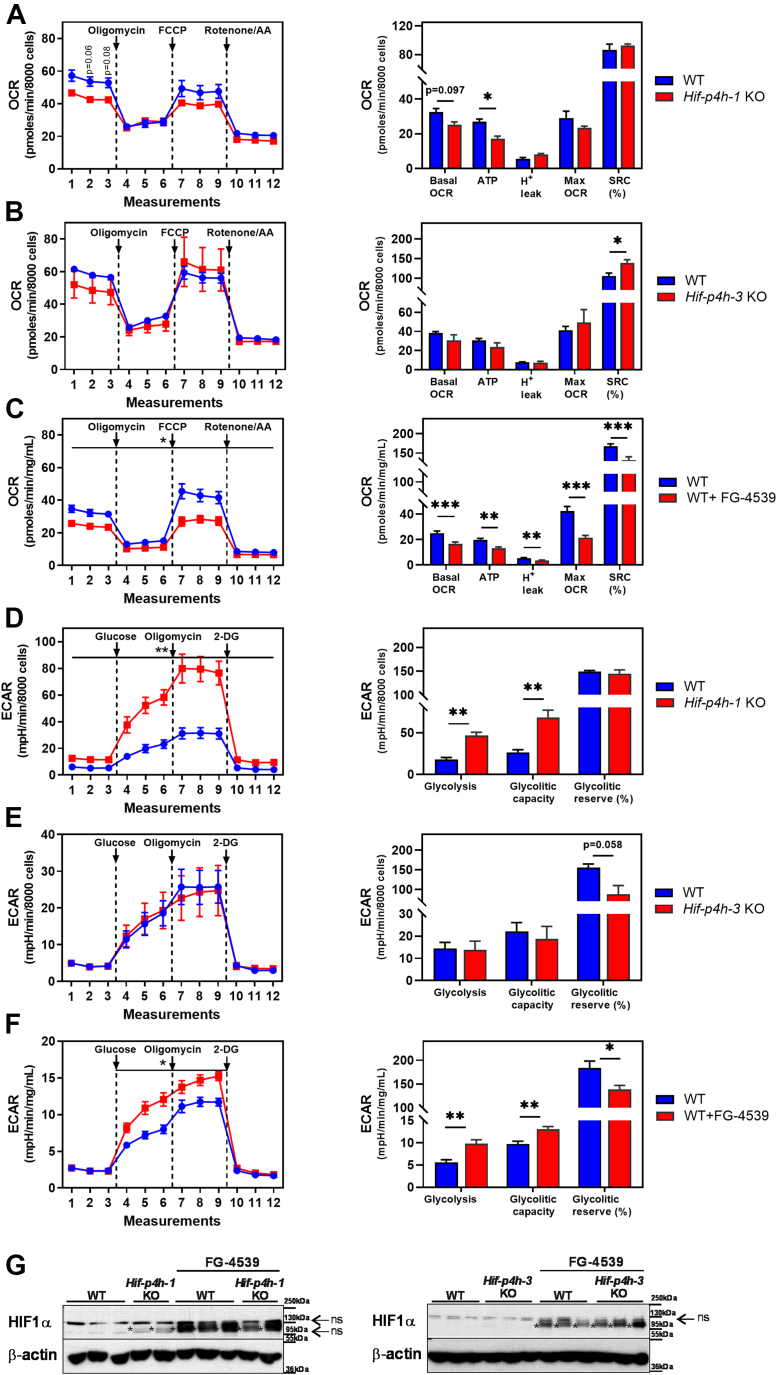
**Metabolic profiles and Western blot analysis for HIF1α of *Hif-p4h-1* KO and *Hif-p4h-3* KO MEFs and wild-type MEFs treated with pan-HIF-P4H inhibitor FG-4539.***A*–*C*, oxygen consumption rate (OCR) at basal level and after consecutive injections of oligomycin (1 μM), FCCP (2 μM), and rotenone/antimycin A (0.5 μM) and quantification of basal respiration, ATP production, proton leak, maximum respiration, and spare respiratory capacity. *D*–*F,* extracellular acidification rate (ECAR) at basal level and after sequential additions of glucose (10 mM), oligomycin A (1 μM), and 2-deoxyglucose (50 μM) and quantification of glycolysis, glycolytic capacity, and glycolytic reserve. *A*–*F*, mean of all independent cell clones measured in triplicate (n = 3 *Hif-p4h-1* WT, n = 1 *Hif-p4h-1* KO*,* n = 3 *Hif-p4h-3* WT, n = 3 *Hif-p4h-3* KO, n = 3 WT, n = 3 WT+FG-4539). Data are means ± SEM. ∗*p* ≤ 0.05, ∗∗*p* <0.01, ∗∗∗*p* < 0.001. 2-DG, 2-deoxyglucose; FCCP, carbonyl cyanide-4-(trifluoromethoxy) phenylhydrazone; OCR, oxygen consumption rate, SRC, spare respiratory capacity. *G*, western blot analysis of MEF HIF1α protein levels. β-Actin was used as a loading control. Positive bands are marked with an *asterisk* (∗). ns, nonspecific.

In order to gain further functional understanding on the contribution of inhibition of the individual HIF-P4Hs to metabolism we studied oxygen consumption and extracellular acidification rates of mouse embryonic fibroblasts (MEFs) generated from the *Hif-p4h-1* and *Hif-p4h-3* strains and WT MEFs treated with FG-4539. We have earlier reported that, in similar analyses, *Hif-p4h-2*^*gt/gt*^ MEFs, which have ∼80% knockdown of *Hif-p4h-2* mRNA and normoxic stabilization of HIF1α, have about 40% reduced oxygen consumption and ATP production and about 30% increased glycolysis compared with WT (13). Our analyses showed significant differences in the basal and maximal oxygen consumption rate, ATP production, and proton leak between the strains; the FG-4539-treated WT MEFs had reduced rates by 35 to 50% compared with controls, the ATP production of the *Hif-p4h-1* KO MEFs was down by about one-third, whereas no differences indicative for decreased OXPHOS between the *Hif-p4h-3* KO and WT MEFs were detected (Fig. 9, *A*–*C*). In line, the spare respiratory capacity was slightly higher (131%) in the *Hif-p4h-3* KO MEFs compared with WT, whereas it was slightly lower (78%) in the FG-4539-treated WT MEFs (Fig. 9, *B* and *C*). The rate of glycolysis and glycolytic capacity were significantly increased by 133 to 262% in the *Hif-p4h-1* KO and FG-4539-treated MEFs compared with controls, whereas no difference was detected in the *Hif-p4h-3* KO MEFs (Fig. 9, *D*–*F*). The glycolytic reserve of the FG-4539-treated cells was ∼25% less than the controls (Fig. 9*F*). Western blot analysis of the HIF1α levels indicated normoxic stabilization of it in the HIF-P4H-1-deficient and the FG-4539-treated MEFs but not in HIF-P4H-3-deficient MEFs (Fig. 9*G*). Altogether these data with the data on the *Hif-p4h-2*^*gt/gt*^ MEFs (13) suggest that normoxic HIF1α stabilization associates with the metabolic switch where oxidative phosphorylation is downregulated and glycolysis is upregulated, and which occurs with genetic HIF-P4H-1 and HIF-P4H-2-deficiency in MEFs and is also obtained with a pharmacologic pan-HIF-P4H inhibitor.

## Discussion

The current pharmacological treatment for metabolic dysfunction consists of a combination of several therapeutics, since multiple drugs are required to target specific conditions, *i.e.*, obesity, dyslipidemia, insulin resistance, and fatty liver disease (22). Preclinical data derived mostly from genetic mouse models have suggested that deficiencies in HIF-P4H isoenzyme 1 (18), and especially isoenzyme 2 (12, 16, 20), mediate several effects that can be beneficial in diseases associated with metabolic dysfunction, whereas loss of HIF-P4H-3 can have both beneficial (17) and adverse (19) effects. Pan-HIF-P4H inhibitors, which antagonize all the isoenzymes equally, have now been approved for the treatment of anemia (https://www.ema.europa.eu/en/medicines/human/EPAR/evrenzo) (7), and the use of such inhibitors developed especially to target the erythropoietic response and erythropoiesis-relevant tissues has, in addition to reversing anemia in patients with CKD, been reported to lower their serum total cholesterol, non-HDL cholesterol, and triglyceride levels (10). These data suggest that beneficial metabolic effects may also be achieved with pan-HIF-P4H inhibitors. Furthermore, individuals experiencing environmental exposure to hypoxia by living at higher altitudes have lower fasting glucose levels and better glucose tolerance than those living close to sea level. Thus, demographic studies have associated living at high altitudes with a lower incidence of obesity and diabetes (23, 24, 25).

HIF-P4Hs are the master regulators of the HIF pathway (3). Many HIF target genes regulate glucose and lipid metabolism, and the HIF response mediates a metabolic reprogramming in which glucose intake (independent of insulin) and non-oxygen-demanding glycolytic metabolism are upregulated but OXPHOS is downregulated (6). This comes at the expense of markedly less ATP being generated per glucose molecule, which in general is considered inefficient and a waste of resources but in view of the need for treating the global obesity epidemic stemming from overeating and general inactivity may be desirable (6). We therefore studied here the effects of two preclinical pan-HIF-P4H inhibitors, FG-4497 and FG-4539, on certain anthropometric and metabolic parameters and the expression of metabolic HIF target genes in WT mice. Our data show that both inhibitors are widely applicable for activating the HIF response in WAT, liver, and skeletal muscle, the key tissues for energy metabolism, and conveying the metabolic interplay. These inhibitors alleviated weight gain, lowered the weight of the WAT and liver, improved glucose tolerance, and lowered serum total cholesterol levels. Of importance, the inhibitors were safe to use; they did not cause hypoglycemia, and they only lowered body weight, adiposity, and HOMA-IR in obese and insulin-resistant mice but not in healthy ones. Moreover, similar effects were obtained with both inhibitors, indicating that neither was inferior to the other.

We also aimed to separate out the contribution of each HIF-P4H isoenzyme to the metabolic parameters studied here, in order to provide specifications on which isoenzyme and which tissue should be targeted to obtain the optimal outcome in the treatment of metabolic dysfunction by means of HIF-P4H inhibition. Although categorical conclusions are difficult to formulate, our data show that HIF-P4H-1 inhibition has quite neutral effects on overall metabolism but can provide protection from aging-associated obesity and hypercholesterolemia. Earlier, HIF-P4H-1 loss has been associated with skeletal muscle metabolism, in that it can lower oxygen consumption by reprogramming glucose metabolism from OXPHOS to a more anaerobic form through activation of a PPARα pathway (26). Although HIF-P4H-1 loss has been reported to impair oxidative muscle performance under healthy conditions, it does provide acute protection for myofibers against lethal ischemia *via* a reduction in oxidative stress (26). The hypoxia tolerance of *Hif-p4h-*1 KO skeletal muscles was mainly mediated by HIF2α and was not seen in *Hif-p4h-3* KO or *Hif-p4h-2* heterozygous mice (26). Our data here, and those published earlier on the *Hif-p4h-2*^*gt/gt*^ mice (12, 16, 27), in which the knockdown of *Hif-p4h-2* varies tissue specifically from >90% in the heart to about 80% in skeletal muscle, 50% in WAT and 40% in liver (16, 21), indicate that, of the three isoenzymes, inhibition of HIF-P4H-2 has the greatest effects on metabolism, which also agrees with the fact that it is the most abundant one (3). HIF-P4H-2 deficiency has been associated with lower body weight but did not alter the weight gain of nonobese 6- to 7-month-old mice. In agreement with earlier data showing HIF-P4H-2 deficiency to be protective against fatty liver disease of all etiologies (12, 28), HIF-P4H-2 inhibition proved here to be associated with lower liver weight, and also with lower serum total and HDL cholesterol levels, the latter being the major lipoprotein in mice, thus differing from the situation in humans (29). Interestingly, our data suggest that pan-inhibition of HIF-P4Hs results in a lesser decline in serum total cholesterol levels than in HIF-P4H-2 deficiency alone, an effect that may well be linked to HIF-P4H-3 inhibition-mediated effects, since, at least upon aging, HIF-P4H-1 loss lowered cholesterol levels while HIF-P4H-3 loss increased them, and we identified downregulation of hepatic *Glut1* mRNA in the *Hif-p4h-3* KO liver in association with higher cholesterol levels. HIF-P4H-2 was the only isoenzyme whose inhibition improved glucose metabolism by lowering fasting glucose levels and improving glucose tolerance in GTT. These data agree with earlier published observations (16, 20). In contrast to HIF-P4H-2, inhibition of HIF-P4H-3 mediated higher body weight and weight gain, higher WAT and liver weights, and higher liver triglyceride levels, especially upon aging. Also, HIF-P4H-3 inhibition resulted in hyperglycemia, higher insulin resistance, and glucose intolerance in GTT. In earlier studies, acute hepatocyte-specific HIF-P4H-3 loss has been shown to improve insulin sensitivity and ameliorate diabetes by specifically stabilizing HIF2α and the upregulation of *Irs2* transcription and insulin-stimulated protein kinase B activation (17). HIF-P4H-3 overexpression has been associated with acceleration of the progression of atherosclerosis (30), while its developmental loss has been associated with protection against abnormal sympathoadrenal development and systemic hypotension (31).

In the present analyses of HIF target mRNA levels in WAT, pan-inhibition of HIF-P4Hs upregulated glucose intake and glycolytic metabolism mRNAs, indicating that WAT metabolism can be targeted *via* pharmacological inhibitors and that inhibition of all the HIF-P4H isoenzymes can contribute to this. Although not reaching significance, there was a trend for HIF-P4H-2 deficiency to be associated with lower adipose *Ccl2* mRNA levels, while HIF-P4H-3 deficiency upregulated these levels. This is in line with the detection of more macrophage aggregates in the WAT of aged *Hif-p4h-3* KO mice and their higher HOMA-IR scores and the fact that adipose tissue inflammation is closely associated with obesity-induced insulin resistance. Despite these negative effects of HIF-P4H-3 loss in WAT, its inhibition, like that of HIF-P4H-1 and HIF-P4H-2, upregulated adipose *Glut1* (*Slc2a1*), and glycolytic mRNAs, suggesting that the counteractive effects may not have been mediated by adipose tissue HIF-P4H-3 inhibition *per se* but may have stemmed from systemic effects. The expression of *Adipoq* mRNA was conversely downregulated in the aged *Hif-p4h-3* KO WAT and upregulated in the vehicle-treated *Hif-p4h-3* KO WAT. One potential explanation for the difference could be the solvent meglumine used in treating these mice, as this has been associated with improved glucose tolerance and limiting long-term weight gain (32).

In the liver both inhibitors upregulated metabolic HIF target mRNAs, especially in the samples collected from animals sacrificed 6 h after the last dose, suggesting fast hepatic drug metabolism. Upregulation of *Glut1* (*Slc2a1*) mRNA, glycolytic mRNAs, and the insulin sensitivity–increasing *Irs2* mRNA was seen in association with HIF-P4H-1 and HIF-P4H-2 inhibition, whereas HIF-P4H-3 inhibition was associated with their downregulation. Despite these similarities between isoenzymes 1 and 2, and the differences between isoenzymes 1/2 and 3, only the inhibition of HIF-P4H-2 led to better glucose tolerance in GTT, suggesting that other mRNAs, and potentially other tissues, contributed to this. Interestingly, the major lipogenic mRNAs in the liver, *Srebp1c,* and its targets *Acca*, *Fasn,* and *Scd1*, which we have reported earlier to be downregulated in 1-year-old *Hif-p4h-2*^*gt/gt*^ mouse livers (16), and partially here, too, were also downregulated in *Hif-p4h-3* KO liver upon aging and when challenged with FG-4497. Considering the higher liver weight and hepatic triglyceride content observed in the aged *Hif-p4h-3* KO mice, it is likely that this downregulation served as feedback to limit further *de novo* lipogenesis.

Also, both inhibitors upregulated metabolic HIF target mRNAs in skeletal muscle, the effect being more widespread in the C57BL6/N/Sv129 background than in C57BL6/N. Glycolytic mRNAs were upregulated following HIF-P4H-1 and HIF-P4H-2 inhibition, and in the case of *Pdk4* mRNA also in *Hif-p4h-3* KO skeletal muscle when treated with FG-4497. Only HIF-P4H-2 inhibition upregulated skeletal muscle *Glut4* (*Slc2a4*) mRNA levels. Even though the regulation of skeletal muscle insulin-dependent glucose intake by GLUT4 mainly occurs at the level of transporter location in the plasma membrane (33), our data show that upregulation of its mRNA levels in the *Hif-p4h-2*^*gt/gt*^ mice is associated with lower fasting glucose levels and better glucose tolerance. Moreover, the mRNAs for *Ppara*, a key regulator of fatty acid oxidation in skeletal muscle, and *Pparg*, the activation of which in skeletal muscle can have a significant protective effect on whole-body glucose homeostasis and insulin resistance (34), were only upregulated in skeletal muscle by HIF-P4H-2 inhibition. Interestingly, upregulation of *Hif-p4h-3* mRNA was observed in *Hif-p4h-1* KO and *Hif-p4h-2*^*gt/gt*^ skeletal muscle. In view of the overall better metabolic outcome achieved in these mouse lines, the above effect could suggest that HIF-P4H-3 loss or inhibition in skeletal muscle is especially detrimental.

Altogether, our data speak for beneficial systemic effects on metabolism achieved by HIF-P4H-2 inhibition, while HIF-P4H-1 inhibition conveys some beneficial effects but is mostly neutral and HIF-P4H-3 inhibition clearly has detrimental effects on glucose and lipid metabolism. Analyses of the data are also complicated by the endogenous feedback loop in the HIF system, in that *Hif-p4h-2* and *Hif-p4h-3* are endogenous HIF target genes (35, 36, 37, 38), *i.e.*, the outcome may have been influenced by these isoenzymes potentially providing compensation for the loss of the catalytic activity of others. Moreover, direct comparison between the data for different mouse lines is not possible. It is very important, as also evidenced here, that comparisons between genotypes should only be made within the same strain and cohort. To overcome the systemic complexity, we studied the rates of OXPHOS and glycolysis in MEFs extracted from the HIF-P4H isoenzyme-deficient mice or WT cells treated with FG-4539. These data, together with our earlier published data on HIF-P4H-2-deficient MEFs (13), supported the *in vivo* findings confirming that the metabolic reprogramming is especially mediated by HIF-P4H-2 inhibition while HIF-P4H-1 inhibition can contribute to it but HIF-P4H-3 inhibition does not. Treatment with the pan-HIF-P4H inhibitor also conveyed the metabolic switch suggesting that inhibition of HIF-P4H-3 on top of HIF-P4H-1 and HIF-P4H-2 cannot revert the phenotype. In all cells, the metabolic reprogramming associated with normoxic HIF1α stabilization. Substrates other than HIFα have been reported to HIF-P4H-3 (38, 39, 40), but their hydroxylation has not been confirmed *in vitro* (41). We cannot therefore conclude here whether the deficiency of HIF-P4H-3 inhibition to stabilize HIF1α and phenocopy metabolic reprogramming was due to it acting on another substrate or something else, for example, the fact that it prefers HIF2α over HIF1α (3). Despite our intention to provide a comprehensive analysis of the contribution of each HIF-P4H isoenzyme to metabolism, some caveats remain. Our data nevertheless clearly support the further development of HIF-P4H inhibitors—preferably ones that are selective for isoenzyme 2 or enzymes ½—that would target the WAT, skeletal muscle, and liver for the treatment of metabolic dysfunction with the aim of limiting the erythropoietic response, especially that mediated by HIF-P4H-2 inhibition.

## Experimental procedures

### Mouse lines

The animal experiments were performed in accordance with protocols approved by the National Animal Experiment Board of Finland (License number ESAVI/8179/04.10.07/2017, 53/2017, OH10). The mice in all the experiments were housed in a standard environment with a temperature of 21 to 22 °C and a 12-h day/night cycle, and their well-being was monitored daily. The mice had access to a standard rodent diet (Teklad 18% protein rodent diet, ENVIGO) and water *ad libitum.* For study-specific setups, see Fig. S1. The generation of the genetically modified mice has been described earlier, the *Hif-p4h-1* KO strain in (42), the *Hif-p4h-2*^gt/gt^ strain in (27), and the *Hif-p4h-3* KO strain in (43).

### Collection and analysis of mouse tissues

The male mice in the baseline studies were weighed at baseline (8 m/o) and at sacrifice (1 year/o), while those in the pharmacological experiments were weighed every week. Weight change was determined with the formula (final weight – baseline weight). All baseline blood (b) and serum (s) samples were obtained from the *vena saphena.* Fasting (f) samples were measured after a 12-h fast, b-Hb was measured using a Hb meter (HemoCue Hb 201+) and glucose with a glucose meter (Contour, Bayer). Serum was separated out by centrifugation at 3000*g* for 20 min at 4 °C. Body weight, gonadal WAT, and liver weight were measured at the moment of sacrifice. Tissues were either snap-frozen in liquid nitrogen or fixed in formalin (4% formaldehyde, VWR) overnight for histological analysis. Blood samples were taken at sacrifice from the *vena cava*.

### Analysis of blood and serum parameters

Insulin values were determined with a Rat/Mouse Insulin ELISA kit (EZRMI-13K; Millipore) and s-total cholesterol, s-HDL cholesterol, and s-triglyceride levels by enzymatic methods (Roche Diagnostics). s-EPO was measured using an R&D Systems Quantikine ELISA kit (MEP00B).

### Analysis of liver glycogen and triglyceride content

Liver glycogen content was analyzed with the Glycogen Assay Kit (Cayman Chemical, Item No. 700480) using ∼100 mg of liver and liver triglyceride content by an enzymatic method (Roche Diagnostics), the absorbances of the colorimetric products being determined with the Infinite M1000 Pro Multimode Plate Reader (Tecan).

### Histological and immunohistological analyses

Formalin-fixed tissues were embedded in paraffin, cut into 5 μm thin sections and stained with H&E. The slides were studied using a Hamamatsu NanoZoomer S60 slide scanner. WAT adipocyte size was measured using a VisioPharm custom APP. The total area of the region of interest was used as a control between samples. Macrophages were counted from H&E-stained WAT slides by selecting five hot spots from the total tissue area and counting the number of adipose cells surrounded by macrophage aggregates at 20× magnification. The positive identity of the macrophage aggregates was confirmed by immunohistological staining for CD68 (ab955, Abcam, 1:100).

### mRNA extraction and PCR analyses

Liver and muscle mRNAs were isolated using TriPure Isolation Reagent (Roche Applied Science) and purified using an E.Z.N.A. Total RNA Kit I (Omega Bio-Tek). For WAT mRNA, the E.Z.N.A. Total RNA Kit II (Omega Bio-Tek) was used. mRNA was transcribed to cDNA using the iScript cDNA Synthesis Kit (Bio-Rad) with 500 ng of mRNA as a template. Quantitative PCR was performed with iTaq SYBR Green Supermix with ROX (Bio-Rad) in a C1000 Touch Thermal Cycler and a CFX96 Touch Real-Time PCR Detection System (Bio-Rad) with the primers shown in Table S1.

### HIF-P4H inhibitors and treatment protocols

For the metabolic studies with pharmacological HIF-P4H inhibitors the mice were fed with a standard rodent diet (Teklad 19% protein extruded rodent diet, ENVIGO) and water *ad libitum.* One of the two pan-HIF-P4H inhibitors FG-4497 or FG-4539 (FibroGen, Inc) or the vehicle was administered orally to the mice 3 times a week (Fig. S1). The FG-4497 used in the pharmacological studies was dissolved in 0.5% sodium carboxymethyl cellulose and 0.1% polysorbate80, which was also used as a vehicle in the comparisons with FG-4497. FG-4539 was dissolved in water and a 1 M meglumine solution, which was likewise used as the vehicle in comparisons with FG-4539. Individual doses were determined each week on the basis of body weight.

### Western blotting

Nuclear and cytosolic fractions were extracted from ∼100 mg snap-frozen livers using the NE-PER kit (ThermoFisher Scientific). Protein lysates were resolved by SDS-PAGE, blotted, and probed with primary antibodies against HIF1α (NB-100479, Novus Biologicals, 1:500), HIF2α (NB100-122, Novus Biologicals, 1:500), β-actin (NB600-501, clone AC-15, Novus Biologicals, 1:5000), and Hdac1 (10E2, Cell Signalling Technology, 1:1000). The secondary antibody, either anti-mouse or anti-rabbit, was conjugated to horseradish peroxidase (Bio-Rad Laboratories, 1:5000). The Pierce ECL system (ThermoFisher Scientific) was used for detection.

### Glucose tolerance test

GTT was performed after treating the mice for 4 weeks with the pan-HIF-P4H inhibitors or vehicle. The mice fasted for 12 h prior to GTT. Animals were anesthetized (s.c.) with fentanyl-midazolam (0.1 ml/10 g), and glucose (1 mg/g) was administered *via* an i.p. injection. B-glucose was measured (Contour, Bayer) from the *vena saphena* at baseline and 15, 30, 60, and 120 min time points. The treatment was continued for 2 weeks after the GTT, and then the mice were sacrificed. The AUC for GTT was calculated by the summary measures method, and the HOMA-IR scores from the fb-glucose and fs-insulin values were calculated by a formula = (insulin (pmol/l))/(glucose (mmol/l))∗156.65.

### Cell culture

Gender-matched MEFs from *Hif-p4h-1* KO and WT mice were already described (42). MEFs from *Hif-p4h-3* KO and WT mice (43) were isolated at E14.5 and immortalized as described (13). MEFs were cultured in Dulbecco's modified Eagle's medium (Sigma-Aldrich) supplemented with 10% fetal bovine serum (Biowest), 1% nonessential amino acids (Sigma-Aldrich), and 1% antibiotic in a standard cell culture incubator.

### Seahorse XFp analysis

Real-time monitoring of oxygen consumption and extracellular acidification rates of *Hif-p4h-1, Hif-p4h-3,* and their corresponding WT MEFs was performed using a Seahorse XFp Analyzer, Seahorse XFp Cell Mito Stress Test Kit, and Seahorse XFp Glycolysis Stress Test Kit (Agilent) as described (13). For the experiments with pan HIF-P4H inhibitor, WT MEFs were treated with either dimethyl sulfoxide as a vehicle or with 50 μM FG-4539 overnight. All the data were normalized to either cell number or total protein concentration. All the assays were analyzed using the Seahorse XF Report Generator software (Wave, Agilent) and the metabolic parameters were derived from calculations based on the manufacturer’s instructions.

### Statistical analyses

Student’s *t* test was used to compare statistical significances between two groups. All data are presented as means ± standard error of the mean (SEM). *p* ≤ 0.05 was considered statistically significant. In the figures statistical significance is indicated by asterisks: ∗ or # = *p* ≤ 0.05, ∗∗ or ## = *p* <0.01, ∗∗∗ or ### = *p* < 0.001, ∗∗∗∗ or #### = *p* < 0.0001. Grubbs’ test was used to determine outliers in univariate data sets. Pearson correlation coefficients with 95% confidence intervals were used to evaluate associations between metabolic parameters and mRNA expression levels.

## Data availability

All the data used are provided in this article.

## Supporting information

This article contains supporting information.

## Conflict of interest

G. W. is an employee and shareholder of FibroGen, Inc, which develops HIF-P4H inhibitors as therapeutics. J. M. owns equity in the company, which supports her research.
