# Supplementary material for: Contribution of HIF-P4H isoenzyme inhibition to metabolism indicates major beneficial effects being conveyed by HIF-P4H-2 antagonism
Source: J Biol Chem. 2022 Jul 1;298(8):102222. doi: 10.1016/j.jbc.2022.102222 (PMC9352911; doi:10.1016/j.jbc.2022.102222)
Supplement: Supporting information [file mmc1.pdf]

Supporting Information

Contribution of HIF-P4H isoenzyme inhibition to metabolism indicates major beneficial effects being conveyed by HIF-P4H-2 antagonism

Joona Tapio[1,*], Riikka Halmetoja[1,*], Elitsa Y. Dimova[1], Joni M. Mäki[1], Anu Laitala[1], Gail Walkinshaw[2], Johanna Myllyharju[1], Raisa Serpi[1,3] and Peppi Koivunen[1,#]

[1]Biocenter Oulu, Faculty of Biochemistry and Molecular Medicine, Oulu Center for Cell-Matrix Research, University of Oulu, Oulu, Finland.[2]FibroGen Inc., San Francisco, CA, USA.[3]Faculty of Medicine, University of Oulu, Oulu, Finland. Biobank Borealis of Northern Finland, Oulu University Hospital, Finland.

*Equal contribution
#Corresponding author: Peppi Koivunen

List of materials included:
Supporting Table 1
Supporting Figures 1-11

Supporting Table 1. Sequences for primers used in qPCR analyses.

**Gene**	**Forward primer (5' → 3')**	**Reverse primer (5' → 3')**
Acaca	GAAGTCAGAGCCACGGCACA	GGCAATCTCAGTTCAAGCCAGTC
Actb	AGAGGGAAATCGTGCGTGAC	CAATAGTGATGACCTGGCCGT
Adipoq	TGTTCCTCTTAATCCTGCCCA	CCAACCTGCACAAGTTCCCTT
Ccl2	CCTGCTGTTCACAGTTGCC	ATTGGGATCATCTTGCTGGT
Fasn	TCCTGGAACGAGAACACGATCT	GAGACGTGTCACTCCTGGACTTG
Gbe1	ACTGCTTTGATGGCTTCCGT	AACCTTGACCCATTCCGTGG
Glut1 (Slc2a1)	TCAAACATGGAACCACCGCTA	AAGAGGCCGACAGAGAAGGAA
Glut2 (Slc2a2)	TTCCAGTTCGGCTATGACATCG	CTGGTGTGACTGTAAGTGGGG
Glut4 (Slc2a4)	ACACTGGTCCTAGCTGTATTCT	CCAGCCACGTTGCATTGTA
Hif-P4h-1 (Egln2)	AGAACTGGGATGTTAAGGTGCAT	GAAAATGAGCAACCGGTCAAAGAG
Hif-P4h-2 (Egln1)	GCGTCCCAGTCTTTATTTAGATA	CTGGGCAACTACAGGATAAAC
Hif-P4h-3 (Egln3)	GCTGGGCAAATACTATGTCAAG	CTTATTCAGGTAGTAGATACAGGTGATACA
Hk1	GAGTCTGAGGTCTACGACACC	CCCACGGGTAATTTCTTGTCC
Irs2	GTAGTTCAGGTCGCCTCTGC	TTGGGACCACCACTCCTAAG
Ldha	GCATGAGCTTGCCCTTGTTGA	GACCAGCTTGGAGTTCGCAGTTA
Lep	GAGACCCCTGTGTCGGTTC	CTGCGTGTGTGAAATGTCATTG
Lpin1	GCTCCCGAGAGAAAGTGGTGGA	GGCTTTCCATTCTCGCAGCTCCT
Lpin2	AGTTGACCCCATCACCGTAG	CCCAAAGCATCAGACTTGGT
Pdk1	AGGATCAGAAACCGGCACAAT	GTGCTGGTTGAGTAGCATTCTAA
Pdk4	AGGGAGGTCGAGCTGTTCTC	GGAGTGTTCACTAAGCGGTCA
Pfkl	TGCAGCCTACAATCTGCTCC	GTCAAGTGTGCGTAGTTCTGA
Ppara	CCTGAACATCGAGTGTCGAATAT	GTTCTTCTTCTGAATCTTGCAGCT
Pparg	GCCCACCAACTTCGGAATC	TGCGAGTGGTCTTCCATCAC
Ppia	GAGCTGTTTGCAGACAAAGTTC	CCCTGGCACATGAATCCTGG
Scd1	TTCTTGCGATACACTCTGGTGC	CGGGATTGAATGTTCTTGTCGT
Sreb1c (Srebf1c)	GAGCCATGGATTGCACATTT	CTCAGGAGAGTTGGCACCTG

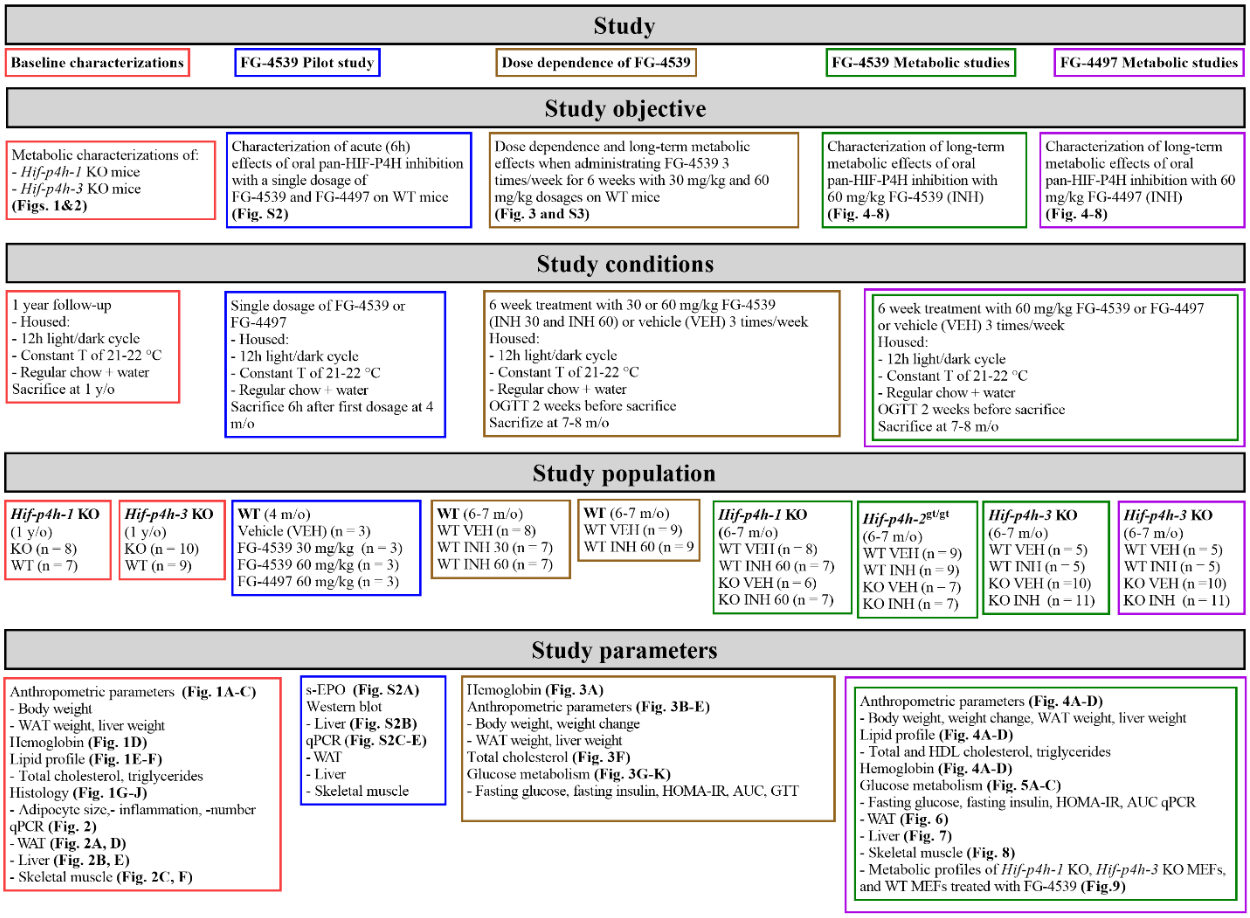

Supporting Figure 1. Flow-chart indicating the populations studied and analyses performed. AUC; area under the curve, qPCR; quantitative polymerase chain reaction, HDL; high-density lipoprotein, HIF-P4H; HIF prolyl 4-hydroxylase, HOMA-IR; homeostatic model assessment for insulin resistance, INH; inhibitor-treated, n; number of mice in the analyses. KO; knockout, m/o; month-old, OGTT; oral glucose tolerance test, s-EPO; serum erythropoietin, VEH; vehicle treated, WAT; white adipose tissue, WT; wild type, y/o; year-old.

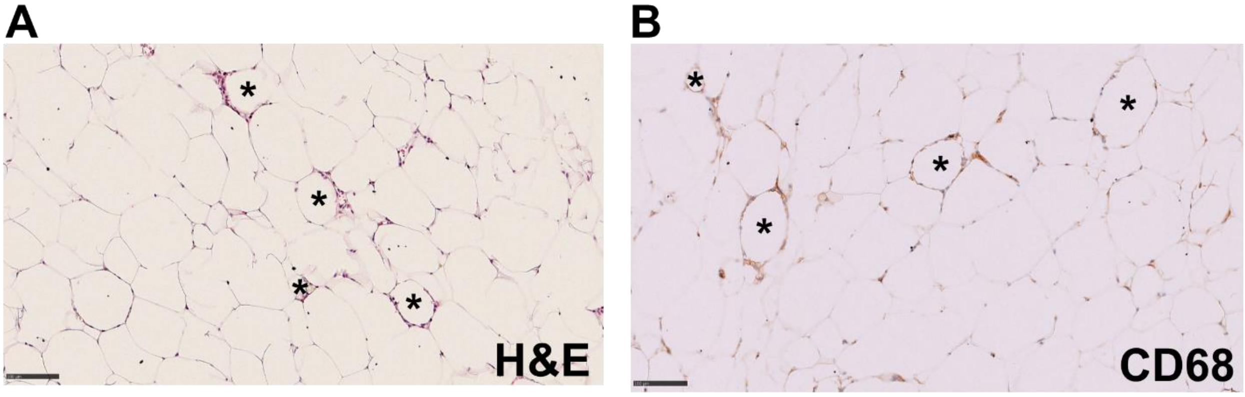

Supporting Figure 2. Comparison of macrophage aggregates in *Hif-p4h-1* KO WAT using H&E (hematoxylin-eosin) and CD68-stained histological slides. Macrophage aggregates are indicated with an asterisk (*). **A** H&E staining of *Hif-p4h-1* KO WAT showing four macrophage aggregates. The image is a reuse of Fig. 1K. **B** CD68 staining (ab955, Abcam, 1:100) of *Hif-p4h-1* KO WAT showing 4 macrophage aggregates. Figures A and B are 20 x magnifications, scare bar length = 100 µm.

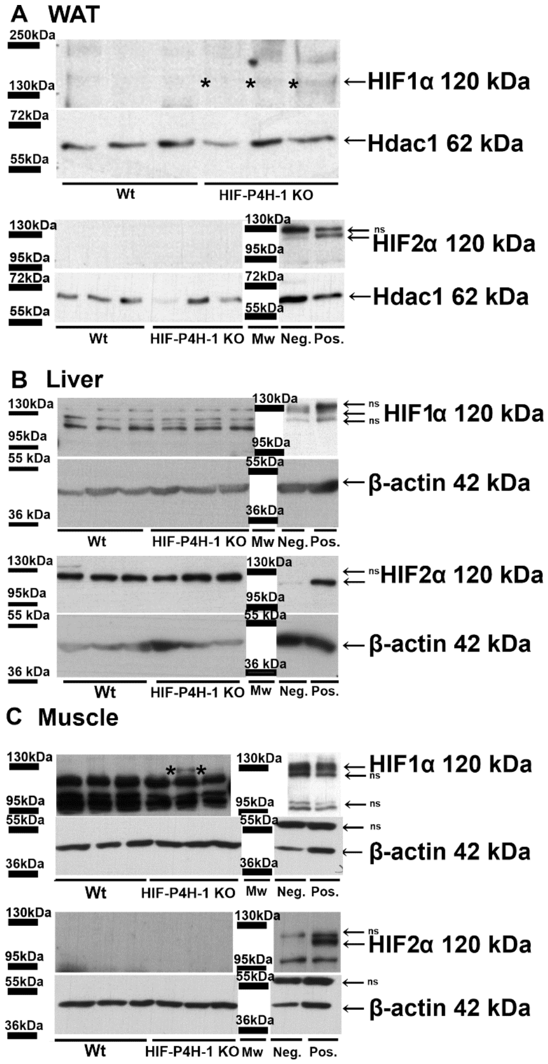

Supporting Figure 3. Western blot analysis of 1-year-old *Hif-p4h-1* KO and WT metabolic tissues. A Western blot analysis of WAT HIF1α and HIF2α protein levels. **B** Western blot analysis of liver HIF1α and HIF2α protein levels. **C** Western blot analysis of skeletal muscle HIF1α and HIF2α protein levels. Positive bands are marked with an asterisk (*). n = 3/group. Mw indicates molecular weight, Neg. indicates a negative control (RCC cells complemented by VHL) and Pos. indicates a positive control (RCC cells). ns indicates non-specific. Histone deacetylase 1 (Hdac 1) and β-actin were used as loading controls.

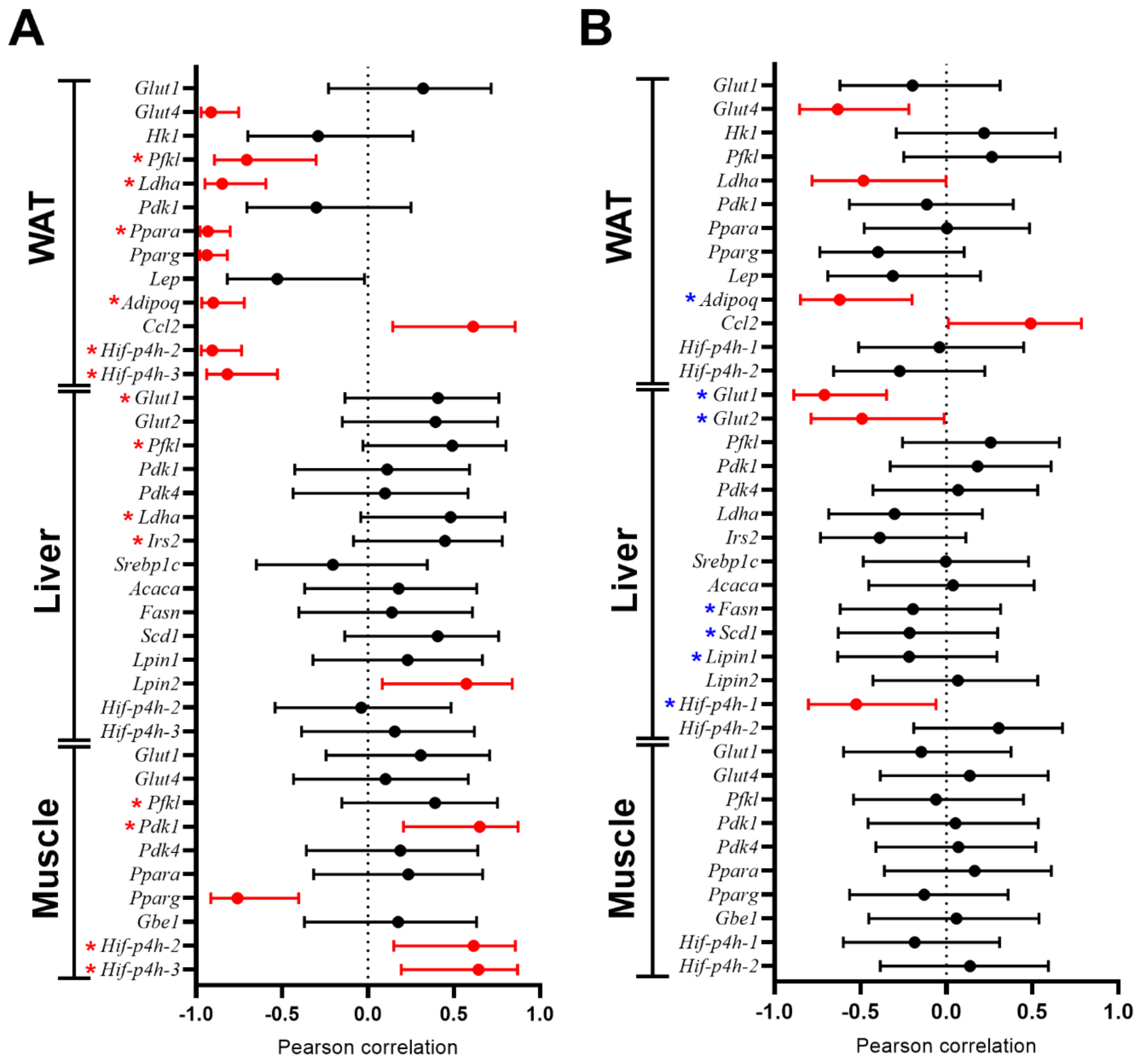

Supporting Figure 4. Pearson correlation coefficients with 95% confidence intervals (CI) representing the associations between body weight and mRNA expression levels in WAT, liver and skeletal muscle of 1-year-old *Hif-p4h-1* and *Hif-p4h-3* KO mice and their C57BL/6N WT littermates. A *Hif-p4h-1* KO mice and their WT littermates. **B** *Hif-p4h-3* KO mice and their WT littermates. A red marker indicates statistical significance, a red asterisk upregulation of the respective mRNA in KO mice relative to WT, and a blue asterisk downregulation of the respective mRNA in KO mice relative to WT. Acaca; acetyl-CoA carboxylase α, Adipoq; adiponectin, Ccl2; chemokine ligand 2, Fasn; fatty acid synthase, Gbe1; 1,4-α-glucan branching enzyme 1, Glut1/2/4; glucose transporter 1/2/4, Hif-p4h-1-3; hypoxia-inducible factor prolyl-4 hydroxylase 1-3, Hk1; hexokinase 1, Irs2; insulin receptor substrate 2, KO; knockout, Ldha; lactate dehydrogenase a, Lep; leptin, Lpin1; lipin-1, Lpin2; lipin-2, Pdk1; pyruvate dehydrogenase kinase, Pdk4; pyruvate dehydrogenase kinase 4, Pfkl; phosphofructokinase l, Ppara; peroxisome proliferator-activated receptor α, Pparg; peroxisome proliferator-activated receptor γ, Scd1; stearoyl-CoA desaturase-1, Screbp1c; sterol regulatory element-binding protein 1, WAT; white adipose tissue, WT; wild type.

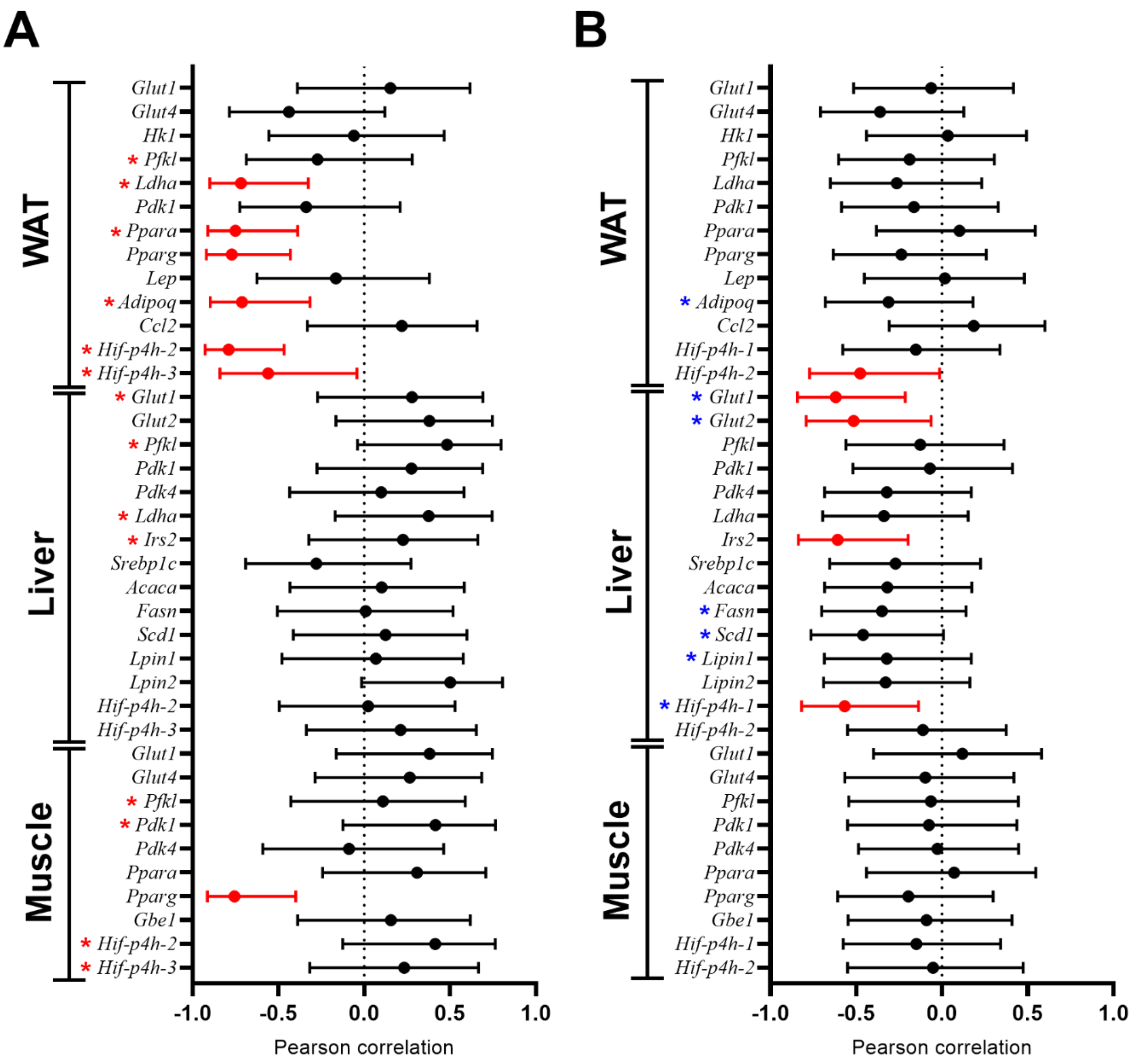

Supporting Figure 5. Pearson correlation coefficients with 95% confidence intervals (CI) representing the associations between WAT weight and mRNA expression levels in WAT, liver and skeletal muscle of 1-year old *Hif-p4h-1* and *Hif-p4h-3* KO mice and their C57BL/6N WT littermates. **A** *Hif-p4h-1* KO mice and their WT littermates. **B** *Hif-p4h-3* KO mice and their WT littermates. A red marker indicates statistical significance, a red asterisk upregulation of the respective mRNA in KO mice relative to WT, and a blue asterisk downregulation of the respective mRNA in KO mice relative to WT. Acaca; acetyl-CoA carboxylase α, Adipoq; adiponectin, Ccl2; chemokine ligand 2, Fasn; fatty acid synthase, Gbe1; 1,4-α-glucan branching enzyme 1, Glut1/2/4; glucose transporter 1/2/4, Hif-p4h-1-3; hypoxia-inducible factor prolyl-4 hydroxylase 1-3, Hk1; hexokinase 1, Irs2; insulin receptor substrate 2, KO; knockout, Ldha; lactate dehydrogenase a, Lep; leptin, Lpin1; lipin-1, Lpin2; lipin-2, Pdk1; pyruvate dehydrogenase kinase, Pdk4; pyruvate dehydrogenase kinase 4, Pfkl; phosphofructokinase l, Ppara; peroxisome proliferator-activated receptor α, Pparg; peroxisome proliferator-activated receptor γ, Scd1; stearoyl-CoA desaturase-1, Screbp1c; sterol regulatory element-binding protein 1, WAT; white adipose tissue, WT; wild type.

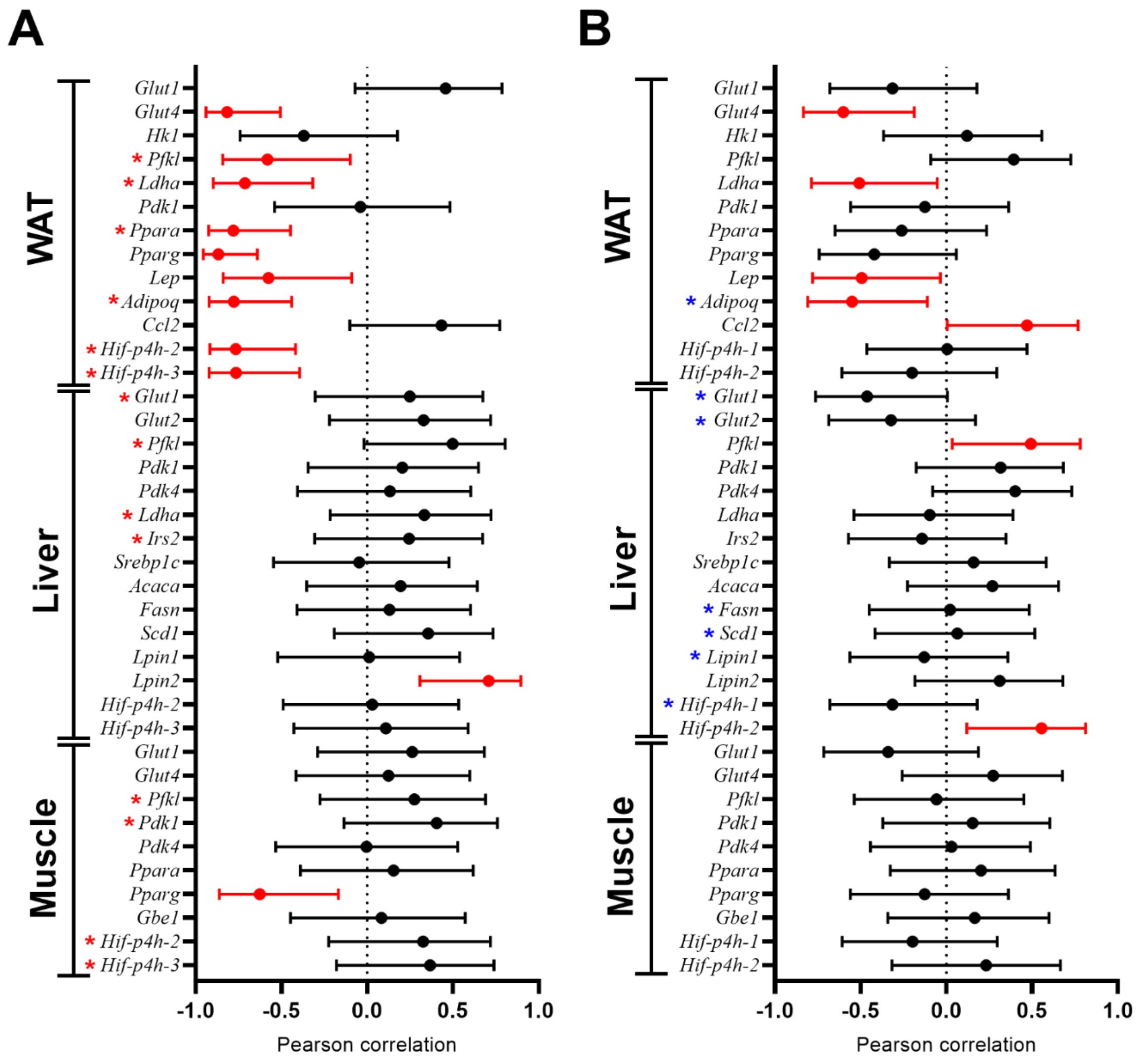

Supporting Figure 6. Pearson correlation coefficients with 95% confidence intervals (CI) representing the associations between liver weight and mRNA expression levels in WAT, liver and skeletal muscle of 1-year old *Hif-p4h-1* and *Hif-p4h-3* KO mice and their C57BL/6N WT littermates. A *Hif-p4h-1* KO mice and their WT littermates. **B** *Hif-p4h-3* KO mice and their WT littermates. A red marker indicates statistical significance, a red asterisk upregulation of the respectivee mRNA in KO mice relative to WT, and a blue asterisk downregulation of the respective mRNA in KO mice relative to WT. Acaca; acetyl-CoA carboxylase α, Adipoq; adiponectin, Ccl2; chemokine ligand 2, Fasn; fatty acid synthase, Gbe1; 1,4-α-glucan branching enzyme 1, Glut1/2/4; glucose transporter 1/2/4, Hif-p4h-1-3; hypoxia-inducible factor prolyl-4 hydroxylase 1-3, Hk1; hexokinase 1, Irs2; insulin receptor substrate 2, KO; knockout, Ldha; lactate dehydrogenase a, Lep; leptin, Lpin1; lipin-1, Lpin2; lipin-2, Pdk1; pyruvate dehydrogenase kinase, Pdk4; pyruvate dehydrogenase kinase 4, Pfkl; phosphofructokinase l, Ppara; peroxisome proliferator-activated receptor α, Pparg; peroxisome proliferator-activated receptor γ, Scd1; stearoyl-CoA desaturase-1, Screbp1c; sterol regulatory element-binding protein 1, WAT; white adipose tissue, WT; wild type.

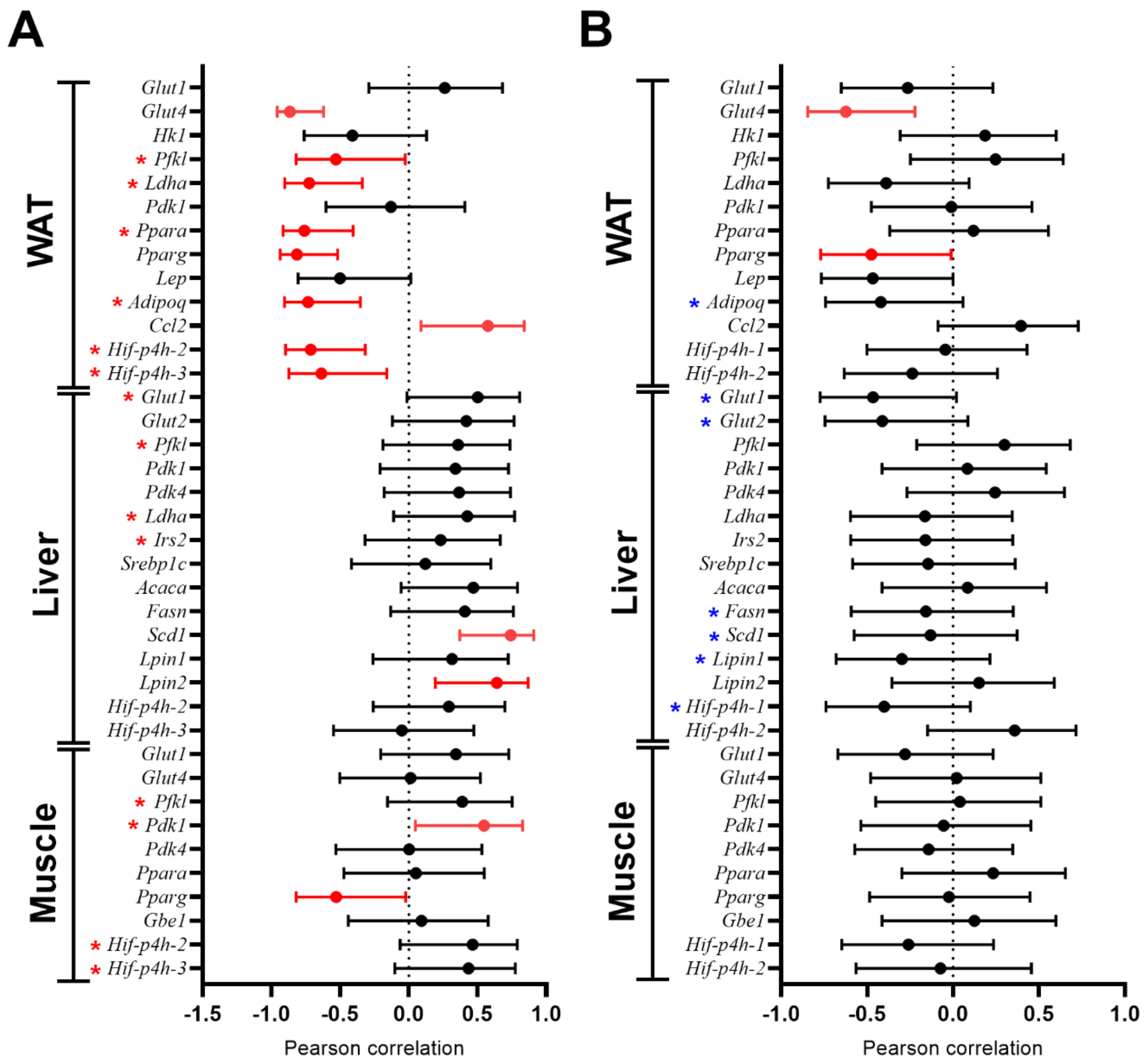

Supporting Figure 7. Pearson correlation coefficients with 95% confidence intervals (CI) representing the associations between liver triglycerides and mRNA expression levels in WAT, liver and skeletal muscle of 1-year old *Hif-p4h-1* and *Hif-p4h-3* KO mice and their C57BL/6N WT littermates. A *Hif-p4h-1* KO mice and their WT littermates. **B** *Hif-p4h-3* KO mice and their WT littermates. A red marker indicates statistical significance, a red asterisk upregulation of the respective mRNA in KO mice relative to WT, and a blue asterisk downregulation of the respective mRNA in KO mice relative to WT. Acaca; acetyl-CoA carboxylase α, Adipoq; adiponectin, Ccl2; chemokine ligand 2, Fasn; fatty acid synthase, Gbe1; 1,4-α-glucan branching enzyme 1, Glut1/2/4; glucose transporter 1/2/4, Hif-p4h-1-3; hypoxia-inducible factor prolyl-4 hydroxylase 1-3, Hk1; hexokinase 1, Irs2; insulin receptor substrate 2, KO; knockout, Ldha; lactate dehydrogenase a, Lep; leptin, Lpin1; lipin-1, Lpin2; lipin-2, Pdk1; pyruvate dehydrogenase kinase, Pdk4; pyruvate dehydrogenase kinase 4, Pfkl; phosphofructokinase l, Ppara; peroxisome proliferator-activated receptor α, Pparg; peroxisome proliferator-activated receptor γ, Scd1; stearoyl-CoA desaturase-1, Screbp1c; sterol regulatory element-binding protein 1, WAT; white adipose tissue, WT; wild type.

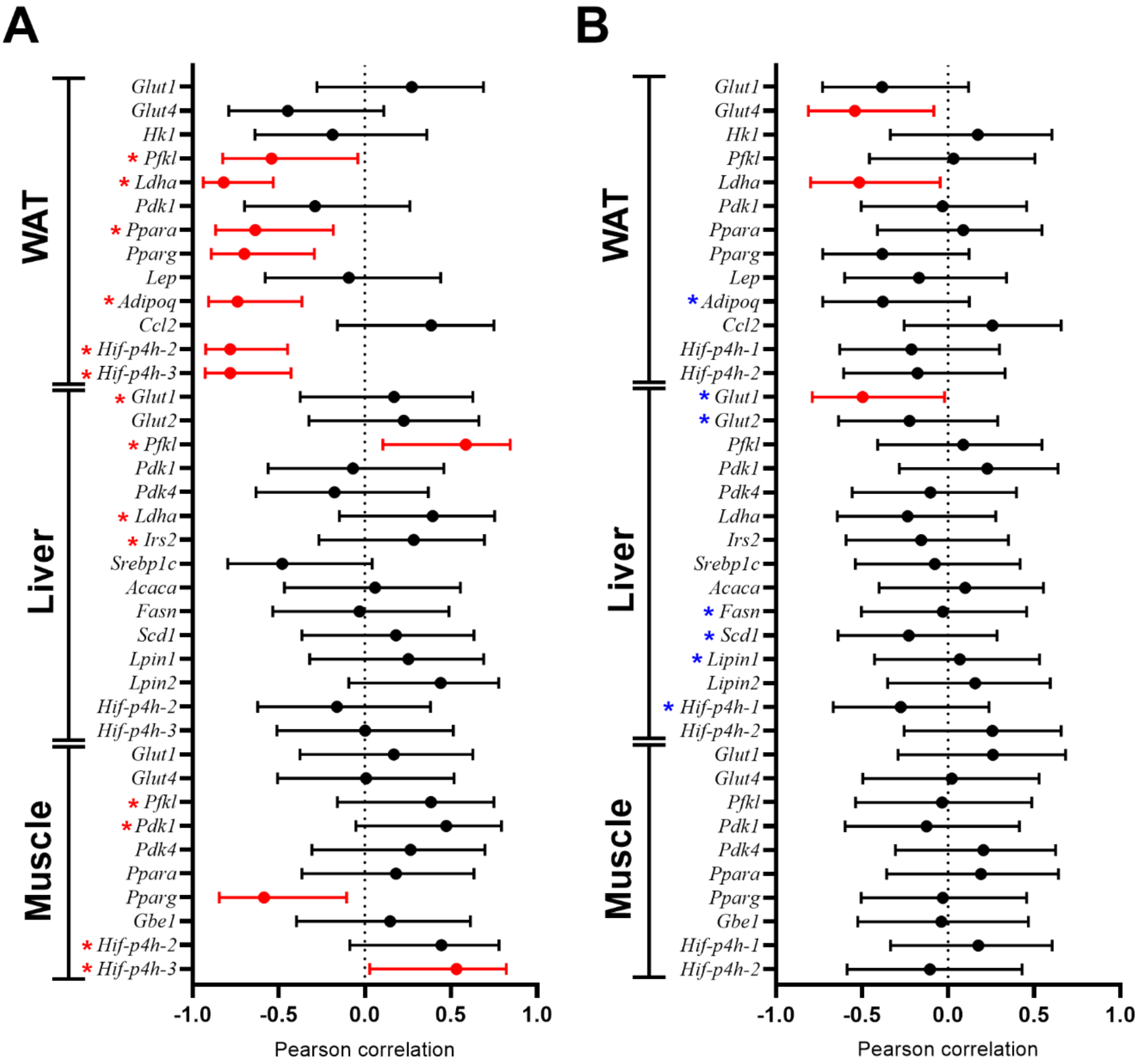

Supporting Figure 8. Pearson correlation coefficients with 95% confidence intervals (CI) representing the associations between total cholesterol levels and mRNA expression levels in WAT, liver and skeletal muscle of 1-year old *Hif-p4h-1* and *Hif-p4h-3* KO mice and their C57BL/6N WT littermates. A *Hif-p4h-1* KO mice and their WT littermates. **B** *Hif-p4h-3* KO mice and their WT littermates. A red marker indicates statistical significance, a red asterisk upregulation of the respective mRNA in KO mice relative to WT, and a blue asterisk downregulation of the respective mRNA in KO mice relative to WT. Acaca; acetyl-CoA carboxylase α, Adipoq; adiponectin, Ccl2; chemokine ligand 2, Fasn; fatty acid synthase, Gbe1; 1,4-α-glucan branching enzyme 1, Glut1/2/4; glucose transporter 1/2/4, Hif-p4h-1-3; hypoxia-inducible factor prolyl-4 hydroxylase 1-3, Hk1; hexokinase 1, Irs2; insulin receptor substrate 2, KO; knockout, Ldha; lactate dehydrogenase a, Lep; leptin, Lpin1; lipin-1, Lpin2; lipin-2, Pdk1; pyruvate dehydrogenase kinase, Pdk4; pyruvate dehydrogenase kinase 4, Pfkl; phosphofructokinase l, Ppara; peroxisome proliferator-activated receptor α, Pparg; peroxisome proliferator-activated receptor γ, Scd1; stearoyl-CoA desaturase-1, Screbp1c; sterol regulatory element-binding protein 1, WAT; white adipose tissue, WT; wild type.

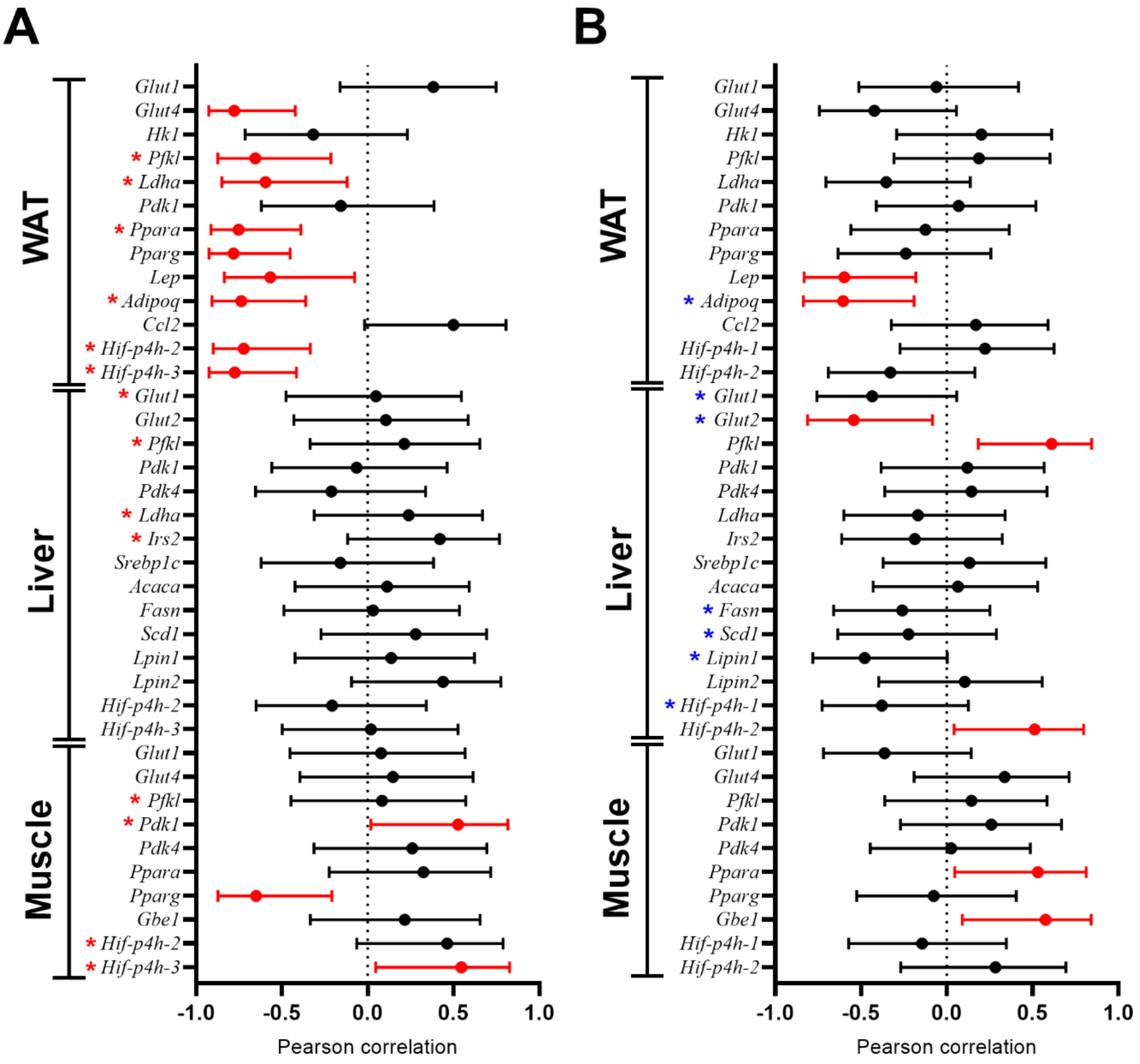

Supporting Figure 9. Pearson correlation coefficients with 95% confidence intervals (CI) representing the associations between the WAT macrophage count and mRNA expression levels in WAT, liver and skeletal muscle of 1-year old *Hif-p4h-1* and *Hif-p4h-3* KO mice and their C57BL/6N WT littermates. A *Hif-p4h-1* KO mice and their WT littermates. **B** *Hif-p4h-3* KO mice and their WT littermates. A red marker indicates statistical significance, a red asterisk upregulation of the respective mRNA in KO mice relative to WT, and a blue asterisk downregulation of the respective mRNA in KO mice relative to WT. Acaca; acetyl-CoA carboxylase α, Adipoq; adiponectin, Ccl2; chemokine ligand 2, Fasn; fatty acid synthase, Gbe1; 1,4-α-glucan branching enzyme 1, Glut1/2/4; glucose transporter 1/2/4, Hif-p4h-1-3; hypoxia-inducible factor prolyl-4 hydroxylase 1-3, Hk1; hexokinase 1, Irs2; insulin receptor substrate 2, KO; knockout, Ldha; lactate dehydrogenase a, Lep; leptin, Lpin1; lipin-1, Lpin2; lipin-2, Pdk1; pyruvate dehydrogenase kinase, Pdk4; pyruvate dehydrogenase kinase 4, Pfkl; phosphofructokinase l, Ppara; peroxisome proliferator-activated receptor α, Pparg; peroxisome proliferator-activated receptor γ, Scd1; stearoyl-CoA desaturase-1, Screbp1c; sterol regulatory element-binding protein 1, WAT; white adipose tissue, WT; wild type.

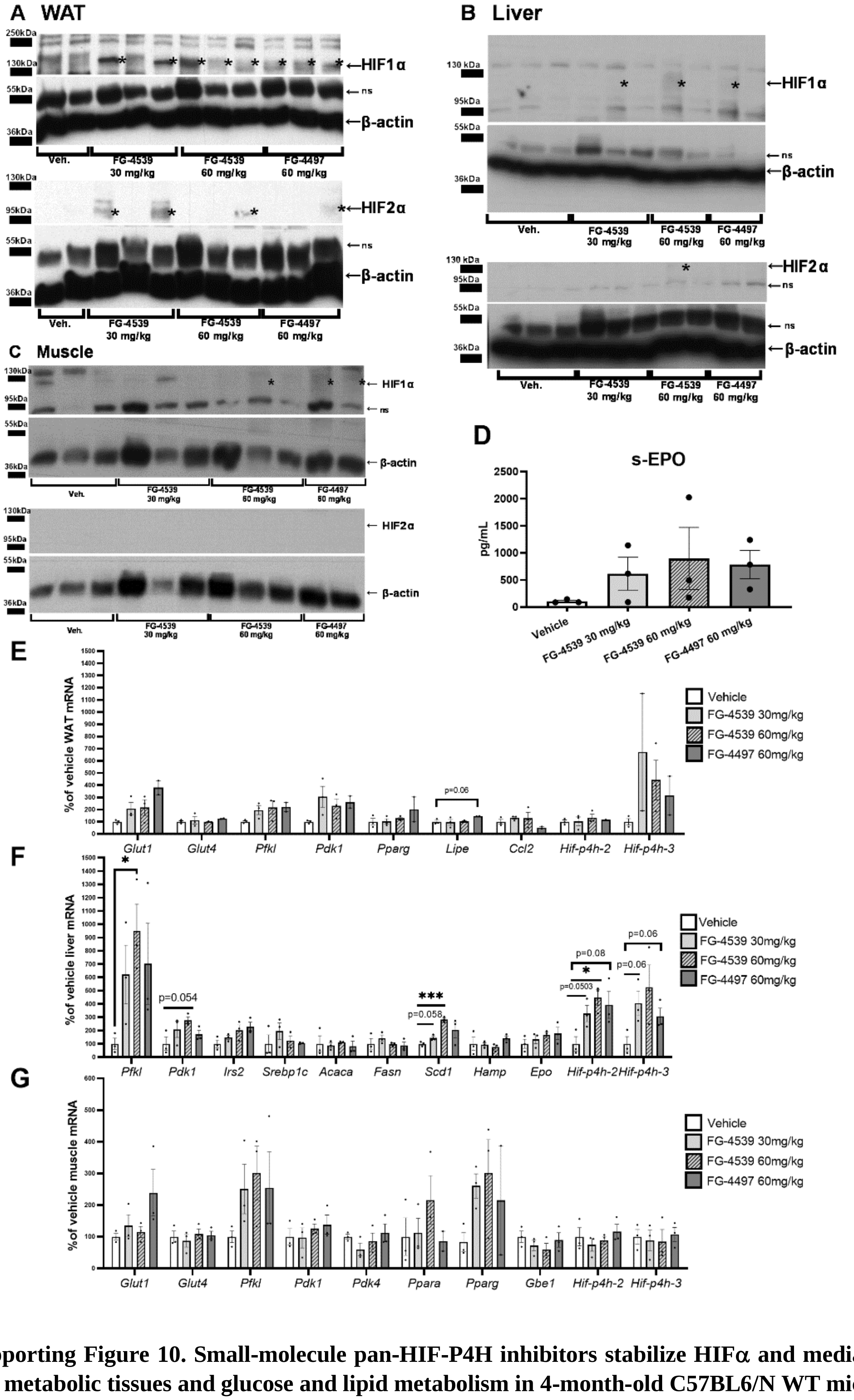

Supporting Figure 10. Small-molecule pan-HIF-P4H inhibitors stabilize HIFα and mediate effects on key metabolic tissues and glucose and lipid metabolism in 4-month-old C57BL6/N WT mice. A Western blot analysis of WAT HIF1α and HIF2α protein levels. **B** Western blot analysis of liver HIF1α and HIF2α protein levels. **C** Western blot analysis of skeletal muscle HIF1α and HIF2α protein levels. **A-C** n = 2-3/group.

Positive bands are marked with an asterisk (*). Pos. indicates a positive control (HIF2α overexpression). ns indicates non-specific. β-actin was used as a loading control. **D** Serum EPO levels (n = 3/group). **E-G** Individual mRNA expressions are presented as percentages relative to the vehicle average (normalized to 100%) of the set mRNA level. **E** qPCR analysis of WAT mRNA levels. **F** qPCR analysis of liver mRNA levels. **G** qPCR analysis of muscle mRNA levels. (n = 3/group). Data are means ± SEM. * $p \leq 0.05$, *** $p < 0.001$. Abbreviations: s, serum; WAT, white adipose tissue, EPO; erythropoietin (protein), Acaca; acetyl-CoA carboxylase α, Fasn; fatty acid synthase, Ccl2; chemokine ligand 2, Gbe1; 1,4-α-glucan branching enzyme 1, Glut1/4; glucose transporter 1/4, Epo; erythropoietin, Hamp; hepcidin, Hif-p4h-2/3; hypoxia-inducible factor prolyl-4 hydroxylase 2/3, Irs2; insulin receptor substrate 2, Lipe; lipase E hormone-sensitive type, Pdk1; pyruvate dehydrogenase kinase, Pdk4; pyruvate dehydrogenase kinase 4, Pfkl; phosphofructokinase l, Ppara; peroxisome proliferator-activated receptor α, Pparg; peroxisome proliferator-activated receptor γ, Scd1; stearoyl-CoA desaturase-1, Screbp1c; sterol regulatory element-binding protein 1.

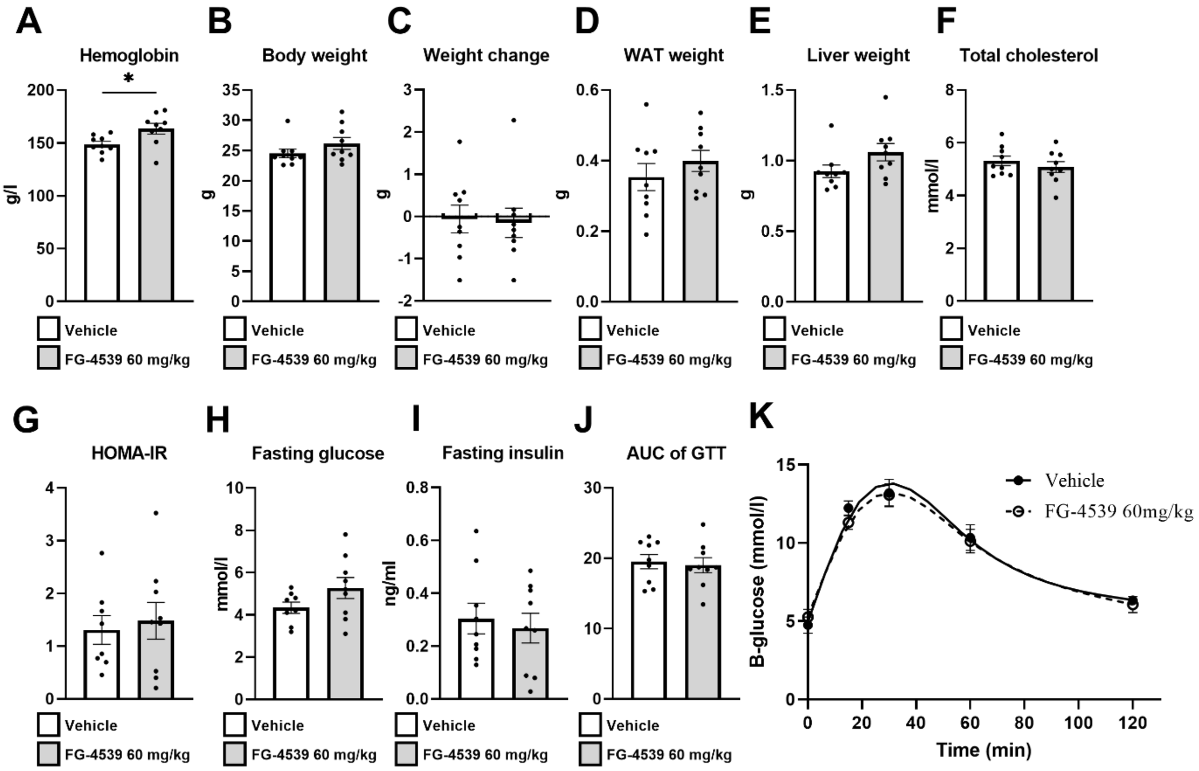

Supporting Figure 11. Effects of long-term administration of a pan-HIF-P4H inhibitor to 7-8-month-old metabolically healthy C57BL/6N/Sv129 WT mice. **A** Hb levels. **B** Body weight. **C** Weight change after four weeks of treatment. **D** Weight of gonadal WAT. **E** Liver weight. **F** Total cholesterol. **G** HOMA-IR. **H** Fasting glucose. **I** Fasting insulin. **J** AUC of GTT. **K** GTT. (n = 9/group). Data are means ± SEM. *$p \leq 0.05$. Abbreviations: AUC; area under the curve, WAT; white adipose tissue; HOMA-IR; homeostatic model assessment of insulin resistance, GTT; glucose tolerance test.
